# *Bacillus velezensis* iturins inhibit the hemolytic activity of *Staphylococcus aureus*

**DOI:** 10.1038/s41598-024-58973-0

**Published:** 2024-04-24

**Authors:** Yasmin Neves Vieira Sabino, Katialaine Corrêa de Araújo Domingues, Paula Mary O’Connor, Pedro Henrique Marques, Eduardo Horta Santos, Marcos Rogério Tótola, Lucas Magalhães Abreu, Marisa Vieira de Queiroz, Paul D. Cotter, Hilario Cuquetto Mantovani

**Affiliations:** 1 Department of Microbiology, Universidade Federal de Viçosa, Viçosa, MG Brazil; 2 Teagasc Food Research Centre, Moorepark, Fermoy, Co. Cork, Cork, Ireland; 3 APC Microbiome Ireland, Cork, Ireland; 4 Department of Microbiology, Immunology and Parasitology, Federal University of Triângulo Mineiro, Uberaba, MG Brazil; 5 Department of Biochemistry and Immunology, Institute of Biological Sciences, Universidade Federal de Minas Gerais, Belo Horizonte, MG Brazil; 6 Department of Fitopathology, Universidade Federal de Viçosa, Viçosa, MG Brazil; 7 Department of Animal and Dairy Sciences, University of Wisconsin-Madison, Madison, WI USA

**Keywords:** Applied microbiology, Bacteria, Bacteriology, Pathogens, Infectious diseases

## Abstract

Bovine mastitis caused by *S. aureus* has a major economic impact on the dairy sector. With the crucial need for new therapies, anti-virulence strategies have gained attention as alternatives to antibiotics. Here we aimed to identify novel compounds that inhibit the production/activity of hemolysins, a virulence factor of *S. aureus* associated with mastitis severity. We screened *Bacillus* strains obtained from diverse sources for compounds showing anti-hemolytic activity. Our results demonstrate that lipopeptides produced by *Bacillus* spp. completely prevented the hemolytic activity of *S. aureus* at certain concentrations*.* Following purification, both iturins, fengycins, and surfactins were able to reduce hemolysis caused by *S. aureus*, with iturins showing the highest anti-hemolytic activity (up to 76% reduction). The lipopeptides showed an effect at the post-translational level. Molecular docking simulations demonstrated that these compounds can bind to hemolysin, possibly interfering with enzyme action. Lastly, molecular dynamics analysis indicated general stability of important residues for hemolysin activity as well as the presence of hydrogen bonds between iturins and these residues, with longevous interactions. Our data reveals, for the first time, an anti-hemolytic activity of lipopeptides and highlights the potential application of iturins as an anti-virulence therapy to control bovine mastitis caused by *S. aureus*.

## Introduction

In 2019, a total of 4.95 million deaths were associated with antimicrobial resistance (AMR) worldwide^1^. *S. aureus* is listed as the second leading pathogen responsible for deaths associated with therapy resistance, only behind *Escherichia coli*^1^. Importantly, *S. aureus* resistant to methicillin was responsible for over 100,000 deaths attributed to AMR in 2019^1^. This species is also a major cause of infections in several animal hosts and because animals can act as a reservoir of staphylococci it can also negatively impact public health^2^.

In livestock production, *S. aureus* is among the main pathogens causing bovine mastitis, one of the most frequent and costly diseases in the dairy sector^3,4^. Antibiotic therapy is currently the main approach to treat bovine mastitis, but the acquisition of resistance by *S. aureus* reinforces the need for new approaches to prevent and control mastitis^5^. Furthermore, the ability of *S. aureus* to invade epithelial cells and form biofilms is correlated with its persistence and difficult eradication from the mammary gland^6^. Therefore, treatments with improved efficacy in eliminating/preventing *S. aureus* colonization are pivotal. In addition, methicillin-resistant *S. aureus* has been listed by the World Health Organization since 2017 in the high priority group for which new therapies should be developed^7^.

Anti-virulence therapy has emerged as a way to decrease bacterial pathogenicity thus preventing/treating infectious diseases while imposing a lower selective pressure on the pathogen toward resistance development^8–10^. The main anti-virulence targets in *S. aureus* are the bacterial membrane, the quorum sensing (QS) system, biofilm formation, and toxin production^11^. In bovine mastitis, toxin production plays an important role in *S. aureus* pathogenicity^12^. A recent study demonstrated that a host-adapted *S. aureus* strain causing chronic mastitis showed increased alpha-hemolysin secretion, which was correlated with an improved ability of *S. aureus* to penetrate and disseminate into udder tissue^13^.

Although efforts are being made in an attempt to block/prevent alpha-hemolysin activity, the potential of bacterial metabolites as anti-virulence compounds is still underexplored. These metabolites can be produced with high yields and have several competitive advantages, including low cost, and low environmental impact, and the biosynthetic genes are often amenable to genetic manipulation allowing improvements in the production process. In this context, *Bacillus* is a bacterial genus easily cultivated in vitro that produces several secondary metabolites, mainly lipopeptides, with a wide range of industrial applications^14,15^.

Lipopeptides produced by *Bacillus* spp. are often classified as surfactins, iturins, and fengycins according to their specific peptide chain and fatty acid structures^14^. In general, these compounds act on microbial surfaces decreasing interfacial tension and disrupting membrane structures, which explains their antimicrobial activity^15^. In addition, some studies have investigated the potential of lipopeptides in impairing bacterial virulence and an earlier study showed that a lipopeptide produced by *Bacillus subtilis* decreased the formation of *S. aureus* biofilms by 90%^16^. More recently, it was demonstrated that *Bacillus* spp. could abolish *S. aureus* colonization in humans. The effect was associated with the production of fengycins by a probiotic *Bacillus* that could eliminate *S. aureus* by inhibiting its quorum sensing system^17^. Quorum sensing also positively upregulates hemolysin production^18^, but studies evaluating the potential of lipopeptides as anti-hemolytic agents are lacking. Here we hypothesize that lipopeptides produced by *Bacillus* spp. could interfere with the production of hemolysins and reduce the hemolytic activity of *S. aureus* strains associated with bovine mastitis. Therefore, this study aimed to identify *Bacillus* spp. with anti-hemolytic activity and characterize the effect of their lipopeptides on the hemolytic activity of *S. aureus*.

## Methods

### Microorganisms and growth conditions

Ninety strains of *S. aureus* were obtained from the Mastitis Pathogens Culture Collection at Embrapa Gado de Leite (Juiz de Fora, Minas Gerais State, Brazil) and used in this study. The bacterial strains were previously isolated from cows with mastitis and identified by using standard biochemical procedures^19,20^. In addition, *S. aureus* O11, a highly hemolytic strain isolated from an ewe with severe mastitis, was included in this study^21^. *S. aureus* strains were cultivated under aerobic conditions in Brain Heart Infusion broth (BHI) or Tryptic Soy Broth (TSB) for 18 h at 37 °C.

Thirty-three cultures of *Bacillus* spp. isolated from various environments (e.g., soil, plants, water, and mastitic milk) in different geographic regions of Brazil (Supplementary Table 1) were used in this study to screen for compounds that could attenuate the hemolytic activity of *S. aureus*. The *Bacillus* spp. strains were cultivated in TSB for 24 h at 30 °C under agitation (200 rpm).

### Hemolysin production

To assess the hemolytic activity of *S. aureus*, the strains were cultivated overnight at 37 °C in a 96-well plate containing BHI broth. The cultures were then transferred to a sheep blood agar base Mueller–Hinton (NewProv, Pinhais, Brazil) using a colony replicator. The inoculated media was incubated at 37 °C for 24 h and then kept overnight at 4 °C. Hemolytic activity in the current study was classified according to the criteria proposed by Da Silva et al.^22^, in which alpha-hemolysin (Hla) production is characterized by colonies showing a transparent zone with complete lysis of erythrocytes after incubation for 24 h at 37 °C (also referred as beta-hemolytic activity) and the production of beta-hemolysin (Hlb) is characterized by presence of an incomplete, non-transparent, lytic zone (also referred as alpha-hemolytic activity) that evolved to complete lysis of the red blood cells after overnight incubation at 4 °C. Experiments were performed with two technical and three biological replicates.

### Interference in hemolysin production

Three *S. aureus* strains characterized as alpha-hemolysin producers were selected for this assay. These isolates and all the bacilli strains tested in the current study were grown in TSB medium. *S. aureus* strains were incubated at 37 °C for 24 h and *Bacillus* spp. was grown at 30 °C for 24 h under agitation. After growth, *Bacillus* supernatants were filtered through a 0.22 µm membrane, and 50 µL of the sterile supernatant was added to 150 µL of TSB broth inoculated with *S. aureus* at an initial OD_600_ of 0.05. The cell suspension was incubated at 37 °C for 24 h. The OD_600_ of the cultures was measured to verify if *Bacillus* supernatants interfered with *S. aureus* growth. The samples were then centrifuged (10,000 g, 10 min, 4 °C) and 100 µL of the supernatants were added to a suspension containing 25 µL of washed sheep erythrocytes diluted into 875 µL of Phosphate Buffer Saline (PBS). The mixture was incubated at 37 °C for 30 min. Following centrifugation (5500 g, 1 min, 4 °C), the OD_543_ of the supernatants was measured to quantify the release of hemoglobin^23^. The hemolytic activity of *S. aureus* assessed in the absence of the supernatants was used as the positive control. We also verified if the compounds produced by *Bacillus* spp. could lyse erythrocytes by evaluating the hemolytic activity of *Bacillus* spp. supernatants alone. Phosphate-buffered saline (PBS) was used as the negative control. Experiments were performed using two technical and two biological replicates.

### Production of lipopeptides by *Bacillus* spp.

For the *Bacillus* spp. strains that demonstrated anti-hemolytic effects, the production of lipopeptides was evaluated over time. The strains were grown in TSB media with an initial OD_600_ of 0.1 for 120 h at 30 °C with agitation (200 rpm). Samples were collected at 0, 3, 6, 12, 24, 48, 72, 96, and 120 h of incubation. The production of lipopeptides was analyzed using the oil displacement assay, with modifications^24^. For this, 20 µL of petroleum was added to 70 mL of distilled water, and 10 µL of the culture supernatants were added to the water/oil interface. The production of lipopeptides was quantified by measuring the oil displacement zone. The cultivation time that resulted in higher production of lipopeptides was selected for the extraction of these compounds.

### Extraction of lipopeptides and evaluation of anti-hemolytic activity

To evaluate if lipopeptides produced by *Bacillus* spp. caused the anti-hemolytic activity against *S. aureus*, the lipopeptides were extracted from *Bacillus* spp. supernatants using the acid precipitation method^25^. Briefly, the cultures with an initial OD_600_ at 0.1 were grown in TSB media at 30 °C, 200 rpm for 48 h. Following a centrifugation step (10,000 g, 15 min, 4 °C), the lipopeptides were precipitated by adjusting the pH of the supernatants to 2.0 using 6 M HCl. The samples were incubated overnight at 4 °C. The acid precipitate was recovered by centrifugation (10,000 g, 15 min, 4 °C) and resuspended in distilled water by neutralizing the pH to 7.0 using 6 M NaOH. The crude extract containing lipopeptides was then lyophilized and a stock solution of 5 mg/mL was prepared and heat sterilized (121 °C/20 min).

The activity of the crude extracts containing lipopeptides against the hemolytic activity of *S. aureus* was evaluated in a range of concentrations made by two-fold serial dilutions (1000–7.31 µg/mL). The three *S. aureus* strains previously selected as well as *S. aureus* O11, a highly hemolytic strain isolated from a severe mastitis case in ewes^21^, were used in this assay. *S. aureus* with an initial OD of 0.05 was grown in 96-well plates (200 µL per well) in the presence of 50 µL of the crude extracts. The samples were incubated at 37 °C for 24 h. After incubation, the plates were centrifuged at 4000 g for 10 min, and 50 µL of the supernatants were added to 200 µL of diluted erythrocytes from sheep blood. Washed erythrocytes from sheep blood were diluted 36× as described previously. The plates were incubated at 37 °C for 1 h to allow the activity of hemolysins and then centrifuged (4000 g/1 min). The hemoglobin released in the supernatant was measured spectrophotometrically at OD_543_. The hemolytic activity of *S. aureus* in the absence of the supernatants was assessed as the positive control and PBS was used as the negative control. The toxic effect of the lipopeptide extracts was also evaluated in different concentrations. The experiment was performed using two technical and two biological replicates.

### Effect of lipopeptide extracts on the expression of genes involved in hemolysin production

*S. aureus* O11 was selected to investigate the effects of lipopeptides on the expression of genes involved in hemolysin production because its genome has been sequenced and the strain shows higher hemolytic activity compared to the bovine mastitis isolates tested in the current study. Moreover, the *Bacillus* spp. lipopeptides showed a similar anti-hemolytic effect against *S. aureus* O11 compared to other *S. aureus* strains isolated from bovine mastitis (Fig. 3). *S. aureus* O11 was cultured in TSB broth containing a crude extract of lipopeptides from one of each *Bacillus velezensis* strains, *B. velezensis* 87 and *B. velezensis* TR47II, at 31.25 µg/mL and 250 µg/mL, respectively. These concentrations were the minimum required to abolish the hemolytic phenotype (100% inhibition of hemolysis). The bacterial cultures were grown for 24 h at 37 °C.

The RNA of *S. aureus* cultures treated or not (control) with the crude extract of lipopeptides was extracted using Trizol Reagent® (Sigma-Aldrich, San Luis, EUA) according to the manufacturer’s instructions. The cell lysis step was optimized by adding 0.1 mm zirconia beads to the tubes followed by shaking for 1 min using a mini bead beater (Biospec Products, Bartlesville, USA). The extracted RNA was treated with DNase (Promega, Madison, USA) to remove any contaminant DNA. The treated RNA was then converted to its complementary DNA using the High-Capacity cDNA Reverse Transcription Kit (Thermo Fisher Scientific, Waltham, USA). All reactions were performed following the manufacturer’s instructions.

To quantify the relative expression of genes involved in hemolysin production, primer pairs were designed for each of the target genes using the Primer3Plus tool (https://www.bioinformatics.nl/cgi-bin/primer3plus/primer3plus.cgi)^26^. The quality of the sequences was verified using OligoAnalyzer (https://www.idtdna.com/pages/tools/oligoanalyzer?returnurl=%2Fcalc%2Fanalyzer). The target genes and primer sequences used in this study are listed in Supplementary Table 2.

The RT-qPCR reactions were performed using the SYBR Green qPCR Master Mix (2×) (BioRad, California, EUA), as described by the manufacturer, using 25 ng/µL of cDNA. The amplification reaction was set to an initial denaturation at 95 °C for 10 min followed by 40 cycles of denaturation at 95 °C for 30 s and annealing and extension at 60 °C for 1 min. The melting curve was performed with a denaturation step at 95 °C for 30 s, initial hold at 60 °C/1 min, followed by an increment in the temperature to 95 °C at a ramp rate of 1 °C/min. All reactions were performed in duplicate with three biological replications.

StepOne Real-Time PCR System (Applied Biosystems, Massachusetts, EUA) was used to perform the RT-qPCR analysis. For each primer, the efficiency curve was plotted using the average Ct and log_10_ of control cDNA concentration. The relative gene expression was calculated based on the equation of the regression line obtained. The 16S rRNA was used as the reference gene.

### Effect of lipopeptides on hemolysin activity

To account for potential post-translational interactions of lipopeptides with *S. aureus* hemolysin, anti-hemolytic activity assays were carried out using cell-free supernatants from *S. aureus*. *S. aureus* O11 was grown in TSB medium for 24 h at an initial OD of 0.05. The culture was centrifuged (10,000 g, 5 min) and the supernatant was treated with lipopeptide extracts from *B. velezensis* 87 and *B. velezensis* TR47II using concentrations varying from 1000 to 125 µg/mL. The samples were incubated at 37 °C for 1 h. Fifty µL of the treated supernatants were then incubated with 200 µL of washed sheep erythrocytes diluted 36× at 37 °C for 1 h. The microplate containing the samples was centrifuged at 4000 g per 1 min. The supernatant was transferred to a new microplate and the hemoglobin released was measured spectrophotometrically at OD_543_.

### Lipopeptide purification

One hundred milligrams of the lipopeptide crude extracts were resuspended in 50 mL of Milli Q water to give a 2 mg/mL solution. This solution was applied to a 5 g (sorbent weight)/20 mL (tube size) C18 solid-phase extraction (SPE) column (Phenomenex, Cheshire, UK) pre-equilibrated with 20 mL methanol and 30 mL of Milli Q water. The column was washed with 40 mL of 25% ethanol and peptides were eluted in 20 mL of 70% 2-propanol plus 0.1% trifluoroacetic acid (TFA), referred here as isopropanol (IPA) and 85% of IPA for extracts from *B. velezensis* 87 and *B. velezensis* TR47II, respectively. The IPA was removed from the C18 SPE IPA eluent by lyophilization (Genevac HT-4X, Genevac Ltd, Ipswich, UK). The samples were applied to a semi-prep Jupiter Proteo RP-HPLC column (250 × 10 mm, 4µ, 90 Å) running a 40–100% acetonitrile 0.1% TFA gradient over 65 min where buffer B was 90% acetonitrile 0.1% TFA. The eluent was monitored at 214 nm and fractions were collected at 1 min intervals. Fractions of interest were pooled and checked for masses of interest using a Bruker Ultraflex MALDI TOF Mass Spectrometry in positive ion reflectron mode. The assignment of the masses obtained to the putative lipopeptides being produced was made by comparison with previous data reported in the literature for lipopeptides produced by *Bacillus* spp.

### Anti-hemolytic activity of purified lipopeptides

Fractions identified as iturins, fengycin, and surfactins were dried and resuspended in methanol to obtain a 5 mg/mL solution. To test the activity of the fractions, *S. aureus* was grown in TSB medium at 37 °C for 24 h. The culture was centrifuged, and the supernatant was incubated with the iturins, fengycins, and surfactins at a final concentration ranging from 1000 to 7.81 µg/mL (two-fold serial dilutions) for 1 h at 37 °C. Fifty µL of the samples were then incubated with 450 µL of sheep blood diluted 36× in PBS and incubated for 1 h at 37 °C to allow the hemolysins to act. The samples were centrifuged at 4000 rpm for 1 min, the supernatant was transferred to a 96-well microplate and the OD_543_ was measured using a Synergy HT spectrophotometer (Biotek, Vermont, USA).

### Oligomerization assay

To evaluate if purified lipopeptides prevent hemolysis by impairing α-hemolysin oligomerization, an SDS-PAGE was performed as described below. The active lipopeptide was mixed with 2.5 µg of alpha-hemolysin (Sigma-Aldrich, St. Louis, USA) at 0.5, 0.25, and 0.125 mg/mL. To promote oligomerization, 5 mM of deoxycholate was added to the samples, and the mixtures were incubated at 22 °C for 20 min^27^. TruPAGE LDS sample buffer 4× (Merck, Darmstadt, Germany) was then added to the samples at 1× final concentration and the mixtures were incubated at 50 °C for 10 min^27^. Twenty-five µL of each reaction mixture was loaded onto NuPAGE 12% Bis–Tris Gel (Invitrogen, Massachusetts, EUA). Ten µL of the SeeBlue Plus2 Pre-stained (Invitrogen, Massachusetts, EUA) were applied as the protein standard. The gel was run at 100 V for 50 min using MES buffer (Invitrogen, Massachusetts, EUA). Gels were stained using the EZblue Gel Staining Reagent (Sigma, Missouri, EUA) overnight.

### Molecular docking

Molecular docking experiments were performed to evaluate if iturins could prevent hemolysis by interacting with the heptameric structure of hemolysin or with its receptor on erythrocytes, known as protein ADAM-10. For ADAM-10 receptor, the E665 residue was chosen because it is considered essential for the binding and consequent activity of hemolysin^28^. Therefore, the grid box was designed to focus on this residue. For hemolysin, three grid boxes were designed to cover the amino acid residues described in the literature to interact with other anti-hemolytic compounds^29–32^. The grid boxes were built using the following amino acid residues: Grid 01—Tyr102, Arg104, Asn105, Ile107, Asp108, Thr109, Glu111, Tyr112, Met113, Ser114, Leu116, Tyr118, Ile142, Gly143 and His144; Grid 02—Gly126, Asp127, Asp128, and Ile132; Grid 03—Asn176, Gln177, Asn178, Trp179, Gly180, Pro181, Tyr182, Gln194, Leu195, Met197, Lys198, Thr199, and Agr200. AutoDockTools v1.5.7^33^ was used to construct the grid boxes, prepare the target protein and convert the file to .pdbqt format for further analysis in Autodock Vina.

Five different structures of iturin A (ligands) with masses corresponding to those found in the MALDI-TOF analysis, were obtained from PubChem in 2D .sdf format. The variants obtained and their characteristics are listed in Supplementary Table 3. The 3D structures were generated using MarvinSketch^34^ and converted to .pdbqt extension using PyRx (https://pyrx.sourceforge.io/). The docking analysis was then performed by Autodock Vina v.1.1.2^35^. Finally, Chimera software version 1.16 was used to visualize the results and generate the final image^36^.

### Molecular dynamics

The complexes composed by the hemolysin heptamer with each of the five iturin A variants were subjected to molecular dynamics simulation using NAMD 3.0b5 package^37^. The systems evaluated were those described in the molecular docking results, including 2 poses discussed for the PubChem CID 101589794, resulting in a total of 6 complexes analyzed. As forcefields, ff19SB was used for the protein, while GAFF was used for the iturins, the latter being parameterized by AMBER antechamber. The systems were generated by AMBER tLEaP^38^, and the complexes were solvated using TIP3P water in a cuboid periodic box with a minimum distance of 15 Å between the complexes atoms and the box edge. These systems were neutralized with NaCl ions. Next, two equilibration phases were applied, firstly using constant-temperature, constant-volume ensemble (NVT—300 K, Langevin) and then using constant-temperature, constant-pressure ensemble (NPT—1.01325 bar, Langevin piston), both being preceded by 1000 steps of minimization and running for 1 ns with harmonic restraints of 1000 kJ/mol/nm placed on solute atoms. These systems, now freed of harmonic restraints, were subjected to 100 ns of production, utilizing the same barostat and thermostat as mentioned. Particle Mesh Ewald (PME) was used for long-range electrostatics. Finally, the trajectory of each system was analyzed using AMBER CPPTRAJ. Analysis included Root Mean Square Deviation (RMSD) calculation of the protein α-carbon atoms and ligand, following fitting to a reference selection. Additionally, Root Mean Square Fluctuation (RMSF) analysis was performed to assess the variability of protein residues compared to the average structure, along with mass-weighted Radius of Gyration (Rg) and monitoring of Hydrogen Bonds (HBs).

### Statistical analyses

The Shapiro–Wilk test was used to assess data normality and the Mann–Whitney test at 95% confidence was applied to make pairwise inferences between treatments and the experimental control. To compare the effect of different concentrations and treatments, two-way ANOVA and Tukey’s multiple comparisons test were used. The statistical analyses were performed using GraphPad Prism software version 8.4.3.

## Results

### Hemolysin production by *S. aureus* strains associated with bovine mastitis

**Figure 1:**
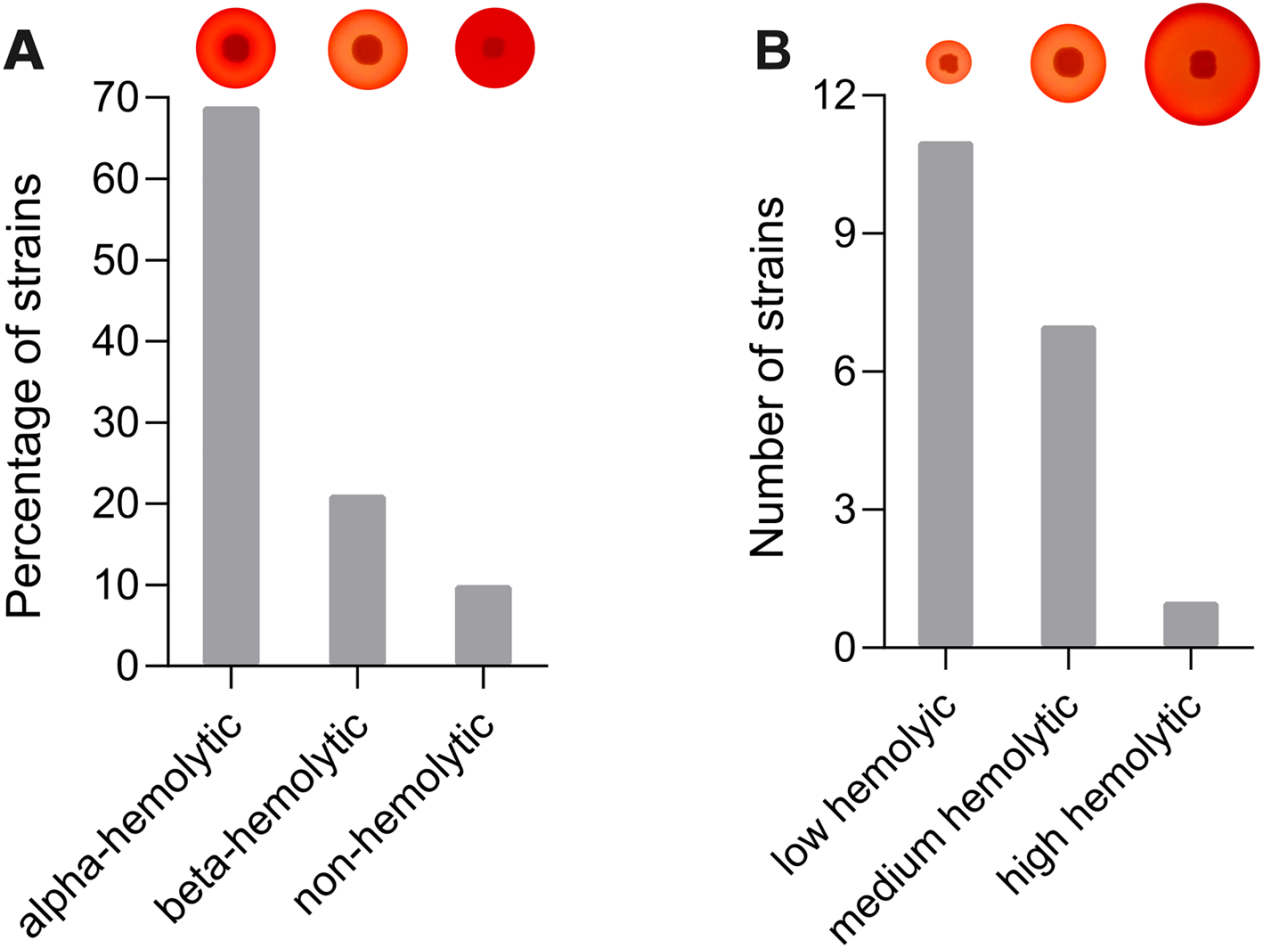
Hemolytic activity of *S. aureus* strains isolated from cows with mastitis on sheep blood agar plates. (**A**) Percentage of strains showing alpha, beta, and non-hemolytic activity. (**B**) Number of beta-hemolytic strains categorized as showing low (< 10 mm), medium (between 10 and 13 mm), and high hemolytic (> 13 mm) activity based on the diameter of hemolysis zones.

The hemolytic activity of *S. aureus* on sheep blood agar plates revealed that most of the *S. aureus* strains tested (n = 62) were alpha-hemolytic, indicated by an incomplete hemolysis around the bacterial colonies after overnight incubation at 37 °C (Fig. 1, Supplementary Fig. 1). Nineteen isolates (~ 21%) were beta-hemolytic and caused complete hemolysis of sheep erythrocytes (Fig. 1A). In the current study, only nine *S. aureus* isolates did not present hemolytic activity on sheep blood agar plates (Fig. 1A). Most of the beta-hemolytic *S. aureus* strains produced a hemolysis zone smaller than 10 mm (Supplementary Fig. 1) and were categorized as having low hemolytic activity (Fig. 1B). *S. aureus* 4051 was the only strain that produced a zone of hemolysis with a diameter greater than 13 mm and, therefore, was classified as having high hemolytic activity.

### Effect of *Bacillus* spp. supernatants on hemolytic activity of *S. aureus*

**Figure 2:**
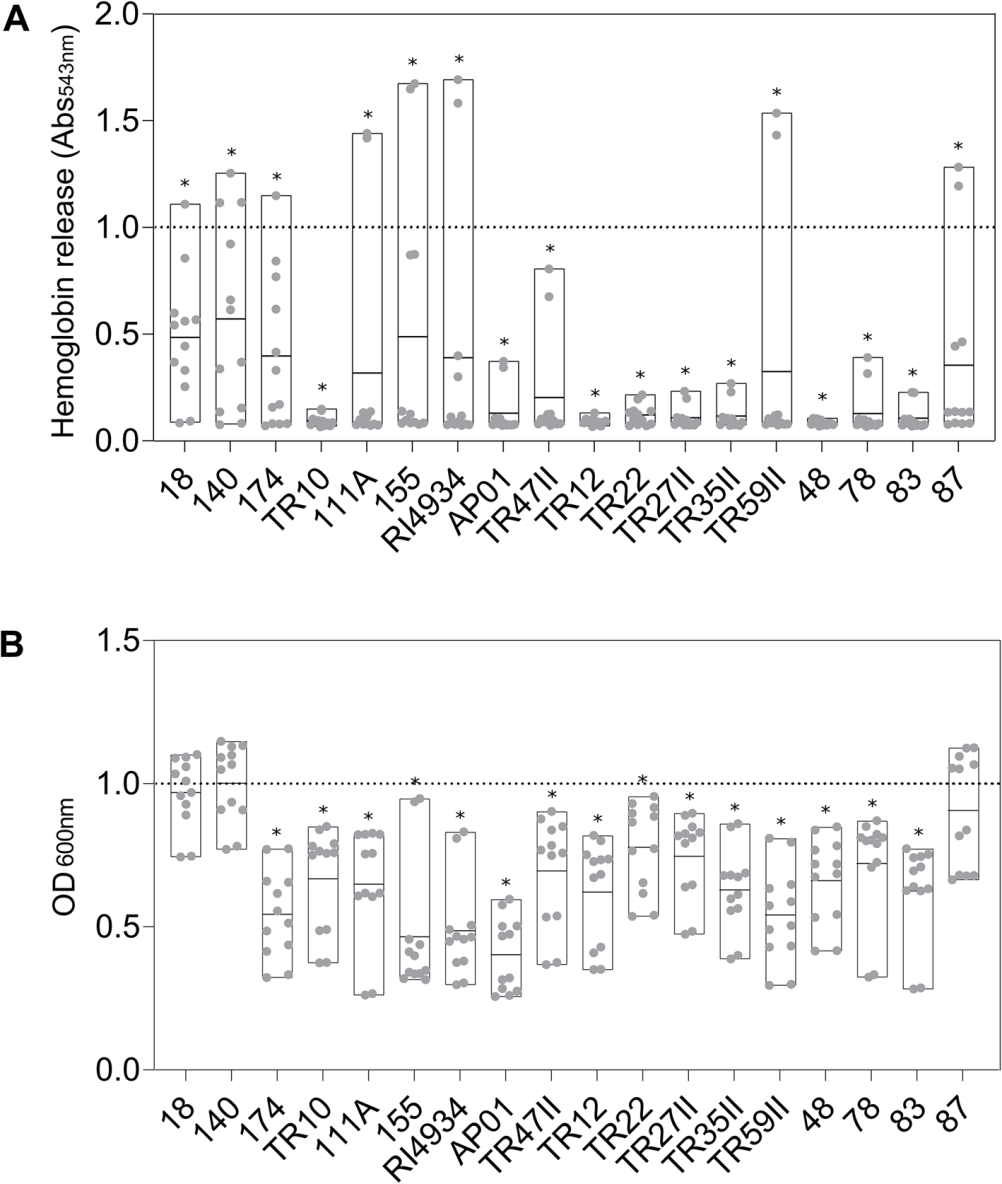
Effect of *Bacillus* spp. supernatants on *S. aureus* hemolysis (**A**) and growth (**B**). The optical density (OD) was normalized in comparison with the controls (set to 1—dotted line). The floating bars show the minimum, maximum, and mean OD of all the replicates of the three *S. aureus* strains analyzed (*S. aureus* 4051, *S. aureus* 4347, and *S. aureus* 4628) represented by gray dots. The line inside the bars represents the mean normalized OD and the asterisks indicate a significant difference in the treatments compared to the control at a 95% confidence level.

Three beta-hemolytic *S. aureus* strains (4051, 4347, and 4628), i.e., producers of alpha-hemolysin^39^, were selected to assess the capacity of supernatants from different *Bacillus* species to reduce the hemolysis of red blood cells. The selected strains showed the highest beta-hemolytic activity among tested cultures. *S. aureus* 4051 was classified, according to the size of the hemolytic zone produced in a sheep blood agar, as having high hemolytic activity while *S. aureus* 4347 and *S. aureus* 4628 were considered to have medium hemolytic activity (Supplementary Fig. 1). Preliminary analyses revealed that the supernatants of 14 *Bacillus* spp. strains analyzed (*Bacillus cereus* 12, *B. cereus* 13, *B. cereus/thuringiensis* 14, *Bacillus* sp. 210, *Bacillus* sp. 221, *Bacillus* sp. 93, *Bacillus* sp. 204, *Bacillus* sp. 201, *Bacillus toyonensis 2*1, *Bacillus altitudinis* 27, *B. cereus/thuringiensis* 32, *B. cereus/thuringiensis* 55, *B. toyonensis* 86, *B. cereus/thuringiensis* 90, and *B. cereus/thuringiensis* 94) were hemolytic against sheep erythrocytes. Therefore, these isolates were excluded from the following analysis. The other strains of *Bacillus* spp. tested reduced the hemolytic activity of *S. aureus* by up to 93% compared to the controls. In contrast, some *Bacillus* strains increased the hemolytic activity of *S. aureus* by up to 28% depending on the strain tested. However, most *Bacillus* cell-free supernatants also affected the growth of *S. aureus,* and only *Bacillus* sp. 18, *Bacillus* sp. 140, and *B. velezensis* 87 reduced the hemolytic activity without inhibiting the growth of *S. aureus* (Fig. 2). The average decrease in hemolytic activity of *S. aureus* strains caused by *Bacillus* sp. 18, 140, and 87 was 52%, 43%, and 65%, respectively (Fig. 2). Moreover, the decrease in hemolytic activity caused by *Bacillus* sp. 18 and *B. velezensis* 87 was considered significant against at least two of the three *S. aureus* strains tested (Supplementary Fig. 2). *Bacillus* sp. 18 reduced the hemolytic activity of *S. aureus* 4051 and *S. aureus* 4628 by 67% and 52%, respectively while *B. velezensis* 87 decreased the hemolytic activity of *S. aureus* 4051 and *S. aureus* 4347 strains by 92% and 86%, respectively (Supplementary Fig. 2). Because some *Bacillus* strains also showed antimicrobial activity against *S. aureus*, their use as a potential source of antimicrobials against bacterial pathogens could be explored in future studies.

### Lipopeptide production and anti-hemolytic activity

To investigate if lipopeptides produced by *Bacillus* spp. were responsible for the anti-hemolytic activity detected in the supernatants, we selected two *Bacillus* strains that showed the highest anti-hemolytic activity without affecting *S. aureus* growth (*Bacillus* sp. 18, *B. velezensis* 87) for further characterization. We also selected a strain of *Bacillus* (*B. velezensis* TR47II) that showed anti-hemolytic activity but also inhibited bacterial growth to evaluate if the antimicrobial activity was due to a “concentration effect” of the lipopeptides released in the supernatant or caused by a distinct bioactive compound produced by *B. velezensis* TR47II. The concentration of lipopeptides in the supernatants of *Bacillus* spp. was higher in the stationary phase and peaked after 48 h of incubation (Supplementary Fig. 3). It should be noted that *B. velezensis* TR47II produces a higher concentration of metabolites with biosurfactant activity, as evidenced by the difference in the diameter of the oil displacement halo compared to the other strains (Supplementary Fig. 3C). This demonstrates a greater capacity of this bacterium to produce lipopeptides in comparison with the other strains of *Bacillus* analyzed in the current study.

**Figure 3:**
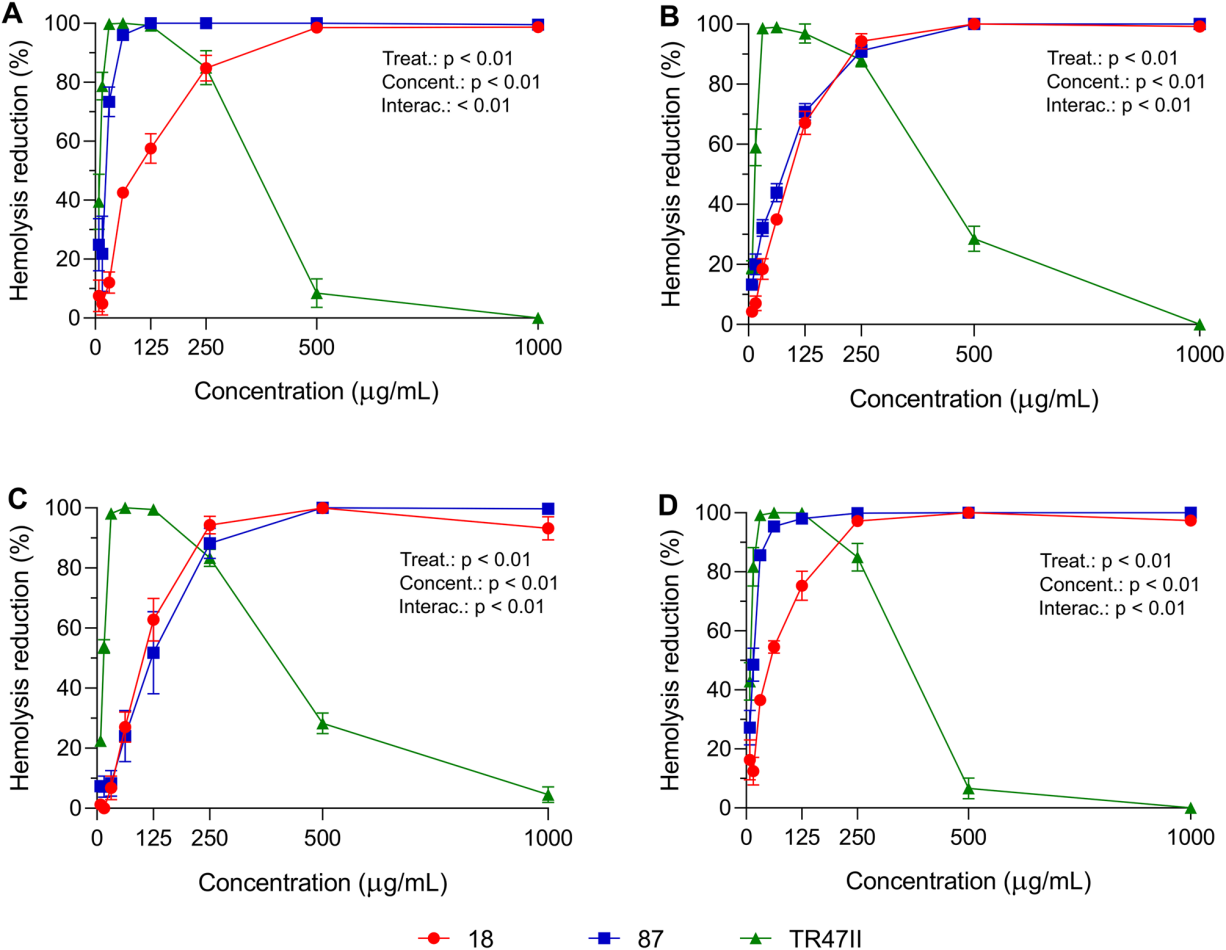
Effect of cell-free crude extracts containing lipopeptides from *Bacillus* spp. on the hemolytic activity of *S. aureus* 4051 (**A**), *S. aureus* 4347 (**B**), *S. aureus* 4628 (**C**), and *S. aureus* O11 (**D**). Each strain of *Bacillus* is color-coded as indicated in the figure legend. Error bars show the standard error of the mean. *S. aureus* cultures without treatment (data set to 100%) were used as controls for data normalization. Models contained fixed effect of lipopeptide extract, concentration, their interaction, and random effect of run. Main effects of concentration and lipopeptide type (*Bacillus* strains) were assessed using two-way ANOVA.

The supernatants of *Bacillus* spp. (48 h, 30 °C, 200 rpm) were subjected to acid precipitation and the anti-hemolytic activity of the crude extract was tested at different concentrations. The concentration of crude extracts containing lipopeptides that reduced the hemolytic activity of *S. aureus* varied according to the strains of *Bacillus* and *B. velezensis* TR47II was the most effective in inhibiting the hemolytic activity of *S. aureus*. The lowest concentration of lipopeptides (7.81 µg/mL) reduced the hemolytic activity of *S. aureus* by more than 40% depending on the test strain and the hemolytic activity of all tested *S. aureus* strains was completely inhibited at 31.25 µg/mL (Fig. 3). *Bacillus* sp. 18 and *B. velezensis* 87 also showed strong anti-hemolytic activity but at higher concentrations (≥ 250 µg/mL) with up to 100% inhibition of *S. aureus* hemolysis (Fig. 3A–D). *B. velezensis* 87 reduced by more than 80% the hemolytic activity of *S. aureus* 4051 and *S. aureus* O11 at 62.5 µg/mL and 31.25 µg/mL, respectively. It was also observed that at high concentrations (≥ 125 µg/mL), the lipopeptides produced by *B. velezensis* TR47II showed hemolytic effects against sheep erythrocytes.

The concentration of lipopeptides significantly (p < 0.001) affected their anti-hemolytic effect against all tested *S. aureus* strains. Lipopeptides produced by different *Bacillus* strains varied significantly in their activity against the tested *S. aureus* strains (p < 0.001 for *S. aureus* strains 4051 and O11, and p = 0.013 for *S. aureus* strains 4347 and 4628). However, multiple comparison analysis showed that depending on the concentration and the *Bacillus* and *S. aureus* strains analyzed, the anti-hemolytic effect of the lipopeptides did not differ statistically (p > 0.05), especially for lipopeptides isolated from *Bacillus* sp. 18 and *B. velezensis* 87.

### Effect of lipopeptide crude extracts from *B. velezensis* strains on the expression of *S. aureus* genes associated with hemolysin production

Real-time PCR experiments were performed to evaluate if the anti-hemolytic activity of lipopeptides was due to a reduced expression of the genes involved in hemolysin production. Because the extracts from *Bacillus* sp. 18 and *B. velezensis* 87 had similar activities in preventing the hemolytic activity of *S. aureus* strains (Fig. 3), and *B. velezensis* 87 was more effective at smaller concentrations, this strain was selected for the qPCR analysis along with *B. velezensis* TR47II. The expression of all hemolysin genes evaluated in this study increased after the treatments with lipopeptide extracts from *B. velezensis* 87 and *B. velezensis* TR47II except for the gene encoding delta hemolysin (*hld*), which showed no difference to the control when cells were treated with extracts from *B. velezensis* TR47II (Supplementary Fig. 4). The quorum sensing genes (*agrA* and *agrC*) also showed increased expression across treatments. In general, the level of expression was higher for treatments with *B. velezensis* 87. Some genes, such as *hla*, *hlg* and *agrC* had increased expression with both lipopeptide extracts, while other genes only increased expression when *S. aureus* was treated with extracts from *B. velezensis* 87 (Supplementary Fig. 4).

### Post-translational anti-hemolytic effect of lipopeptides

Most genes involved in hemolysin production evaluated in the current study showed an increase in expression when *S. aureus* cells were treated with lipopeptide extracts. Therefore, we hypothesized that the anti-hemolytic activity of the lipopeptides produced by *Bacillus* strains was due to direct inhibition of hemolysin activity, not biosynthesis. When *S. aureus* O11 supernatants were treated with lipopeptides produced by *B. velezensis* 87 at 500 µg/mL and *B. velezensis* TR47II at 125 µg/mL, the hemolytic activity of *S. aureus* O11 decreased approximately 90% and 83%, respectively (Supplementary Fig. 5).

### Lipopeptide purification

**Figure 4:**
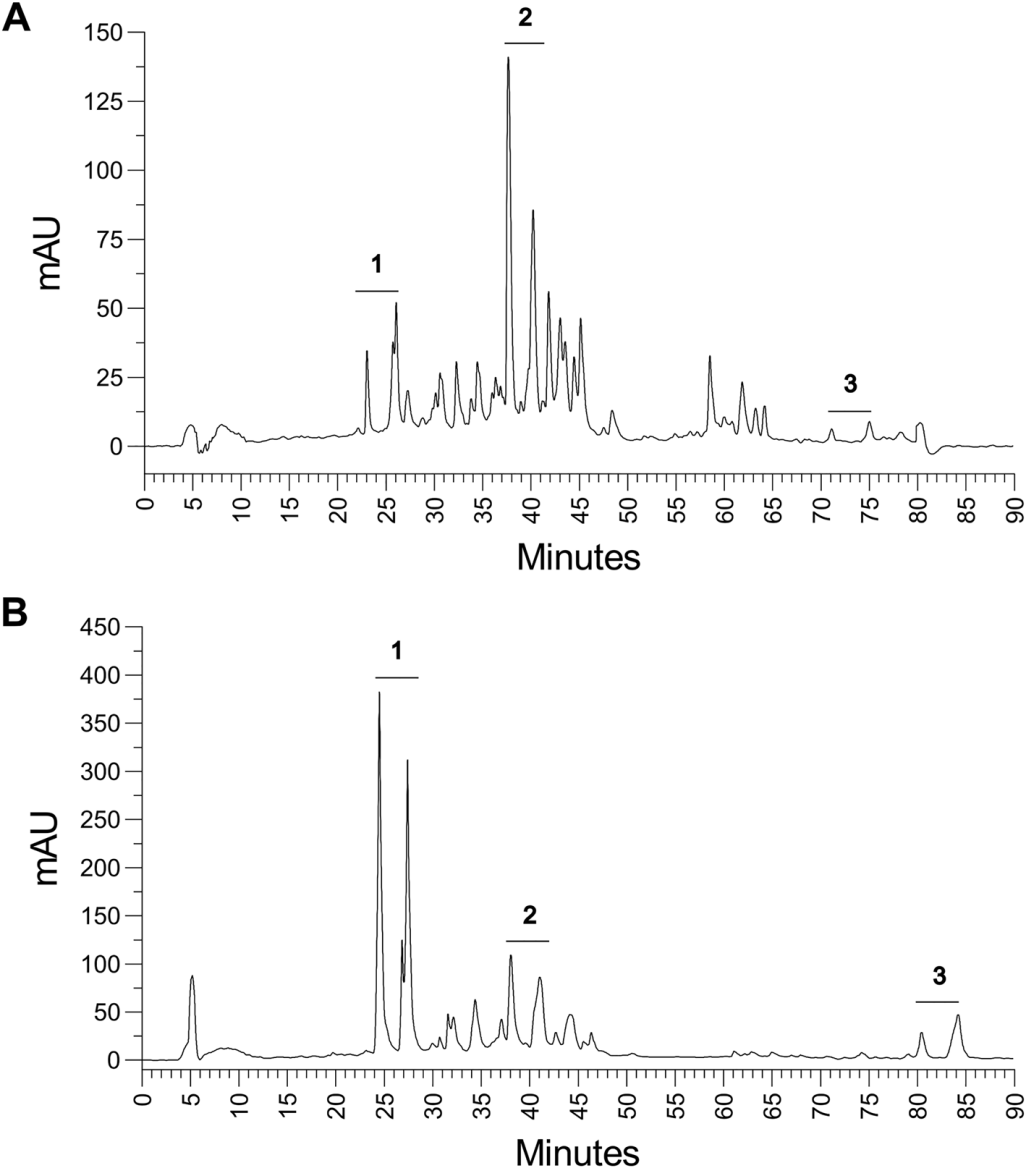
Chromatogram of lipopeptides produced by (**A**) *B. velezensis* 87 and (**B**) *B. velezensis* TR47II analyzed in this study. Numbers 1, 2, and 3 show the two major peaks of iturins, fengycins, and surfactins, respectively. The peptides were analyzed in a C12 chromatography column and eluted using a gradient of acetonitrile + TFA 0.1%.

Lipopeptides produced by *B. velezensis* 87 and *B. velezensis* TR47II were extracted using C18 SPE and further purified and fractionated using reversed-phase chromatography (Fig. 4). Compounds eluting at the beginning of the chromatogram (fractions 23–26) were identified as iturins, while compounds eluting around 38–41 min were fengycins and very hydrophobic compounds eluting with 90% acetonitrile were identified as surfactins by MALDI-TOF Mass Spectrometry. Fractions eluted between 58 and 64 min for *B. velezensis* 87 (Fig. 4A) were analyzed using mass spectrometry, but no compounds could be identified based on the masses detected. *B. velezensis* 87 produced a higher concentration of fengycins while extracts obtained from *B. velezensis* TR47II showed a higher concentration of iturins (Fig. 4). The two major HPLC peaks corresponding to the different classes of lipopeptides were collected and concentrated individually for the in vitro assays. The MALDI-TOF results showed that *B. velezensis* 87 and *B. velezensis* TR47II produce different variants of iturins, with *B. velezensis* 87 potentially producing Iturin A and *B. velezensis* TR potentially synthesizing bacillomycins (Supplementary Figs. 6 and 7, Table 1). Otherwise, the fengycins and surfactins produced by both strains were virtually identical (Supplementary Figs. 6 and 7, Table 1). It should also be noted that more than one peak in the same chromatogram can represent different chemical species (e.g., adducts of H^+^, K^+^, Na^+^) of the same compound (Table 1).

**Table 1:** Masses of lipopeptides purified using reverse phase HPLC.

*B. velezensis* 87			
Fraction	Main peaks	Putative assignments	References
23	1029.0	Iturin A C13 [M+H]+	^40^
	1043.7	Iturin A C14 [M+H]+	
	1081.7	Iturin A C14 [M+K]+	
26	1057.7	Iturin A C15 [M+H]+	
	1079.7	Iturin A C15 [M+Na]+	
	1095.7	Iturin A C15 [M+K]+	
38	1463.7	Fengycin C16 [M+H]+	^41^
41	1477.7	Fengycin C17 [M+H]+	
	1491.7	Fengycin C18 [M+H]+	^42^
71	1022.8	Surfactin C14 [M+H]+	^40^
	1044.8	Surfactin C14 [M+Na]+	
	1060.8	Surfactin C14 [M+K]+	
75	1058.8	Surfactin C15 [M+Na]+	^43^
	1074.8	Surfactin C15 [M+K]+	

*B. velezensis* TR47II			
Fraction	Main peaks	Putative assignments	References
23	1021.7	Bacillomycin L C14 [M+H]+	^44^
	1043.7	Bacillomycin L C14 [M+Na]+	
	1059.7	Bacillomycin L C14 [M+K]+	
26	1035.8	Bacillomycin L C15 [M+H]+	
	1057.7	Bacillomycin L C15 [M+Na]+	
	1073.7	Bacillomycin L C15 [M+K]+	
38	1463.8	Fengycin C16 [M+H]+	^41^
41	1477.7	Fengycin C17 [M+H]+	
	1491.8	Fengycin C18 [M+H]+	^42^
81	1044.8	Surfactin C14 [M+Na]+	^40^
	1060.7	Surfactin C14 [M+K]+	
85	1058.8	Surfactin C15 [M+Na]+	^43^
	1074.7	Surfactin C15 [M+K]+	

### Activity of fengycins, iturins and surfactins

**Figure 5:**
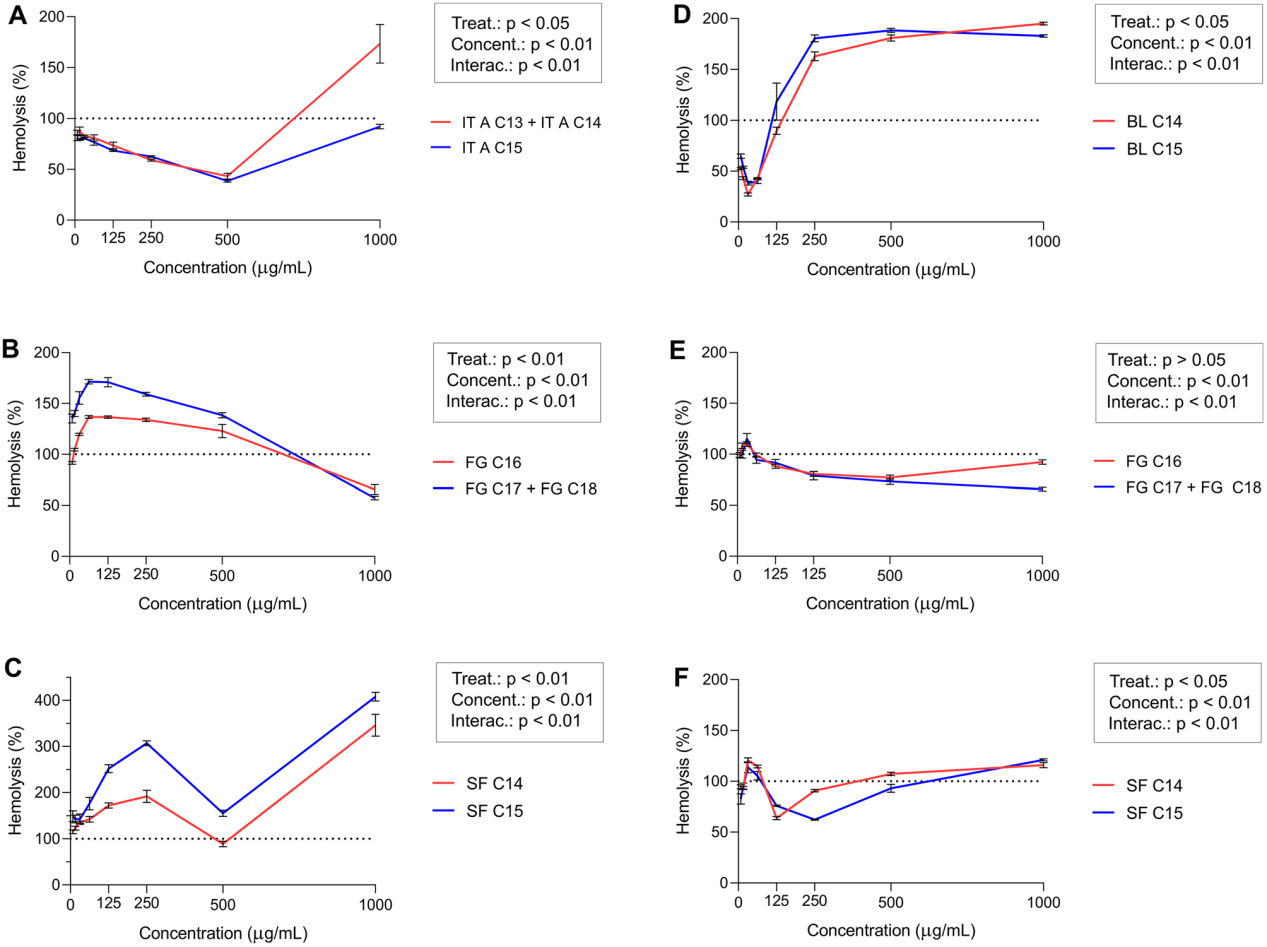
Hemolytic activity of *S. aureus* O11 supernatants in the presence of purified lipopeptides from *B. velezensis* strains. The panel shows the activity of iturins (IT—**A**) and bacillomycins (BL—**D**), fengycins (FG—**B** and **E**), and surfactins (SF—**C** and **F**), produced by *B. velezensis* 87 (**A**–**C**) and *B. velezensis* TR47II (**D**–**F**). The lipopeptides were purified using RP-HPLC and their masses were confirmed using MALDI-TOF MS. The hemolytic activity of the samples was normalized by setting the hemolytic activity of non-treated *S. aureus* O11 supernatant to 100% (dotted lines). Models contained fixed effect of lipopeptide extract, concentration, their interaction, and random effect of run. Main effects of concentration and lipopeptide type (*Bacillus* strains) were assessed using two-way ANOVA.

Among the lipopeptides produced by the *B velezensis* strains analyzed in this study, iturins showed the highest anti-hemolytic activity. Iturins produced by *B. velezensis* 87 decreased the hemolytic activity of *S. aureus* from 12 to 61% depending on the concentration of lipopeptides (Fig. 5A). The iturins produced by *B. velezensis* TR47II were more effective at lower concentrations, reducing the hemolytic activity of *S. aureus* by up to 76% at 31.25 µg/mL (Fig. 5D). However, a higher hemolytic activity was observed above 125 µg/mL for the 1057.74 Da variant and ≥ 250 µg/mL for the 1043.67 Da iturin variant (Fig. 5D). Fengycins produced by *B. velezensis* 87 and *B. velezensis* TR47II only displayed anti-hemolytic activity at 1000 µg/mL and above 62.5 µg/mL, reaching a maximum of 43% and 41% reduction in hemolysis, respectively (Fig. 5B and E). Surfactins produced by *B. velezensis* 87 did not prevent hemolysis by *S. aureus* O11, with only surfactin 1044.82 Da showing an 11% reduction at 500 µg/mL (Fig. 5C). In contrast, surfactins purified from *B. velezensis* TR47II showed anti-hemolytic activity at several concentrations, with the 1058.78 Da variant causing a 38% reduction in *S. aureus* hemolysis at 250 µg/mL (Fig. 5F).

The hemolytic activity of *S. aureus* O11 was significantly affected (p < 0.0001) by different concentrations of iturins, fengycins, and surfactins produced by *B. velezensis* 87 and *B. velezensis* TR47II. Considering all concentrations tested, the two variants of lipopeptides within each lipopeptide class also significantly differ in activity, except for fengycins produced by *B. velezensis* TR47II (p = 0.188).

### Oligomerization assay

SDS-PAGE analysis showed that iturins produced by both *B. velezensis* 87 and *B. velezensis* TR47II did not affect α-hemolysin oligomerization at any of the concentrations tested, as confirmed by the presence of hemolysin heptamers (higher molecular weight band) in all the treatments. This was also demonstrated by the same pattern of bands obtained in the positive control (hemolysin alone) and the treatments and the absence of bands in the negative controls (treatment alone) (Supplementary Fig. 8).

### Molecular docking

Iturin A variants were the lipopeptides most associated with the anti-hemolytic effect of *B. velezensis* against *S. aureus*. Therefore, these compounds were selected for docking analysis. The molecular docking of the iturin variants to the ADAM-10 receptor, present on the erythrocyte membrane, predicted that all iturin structures can form hydrogen bonds with Glu 665. Nonetheless, the binding affinity was low, ranging from − 2.7 to − 2.9 kcal/mol for the best models obtained from Autodock Vina (Supplementary Table 4). The variants could form 3 to 4 hydrogen bonds with ADAM-10, 2 of them with the important residue Glu 665 for iturin A, iturin A1, and iturin A2. Iturin A C-15, in turn, showed the best binding affinity (− 2.9 kcal/mol) with 4 hydrogen bonds with ADAM-10, being one with Glu 665.

**Figure 6:**
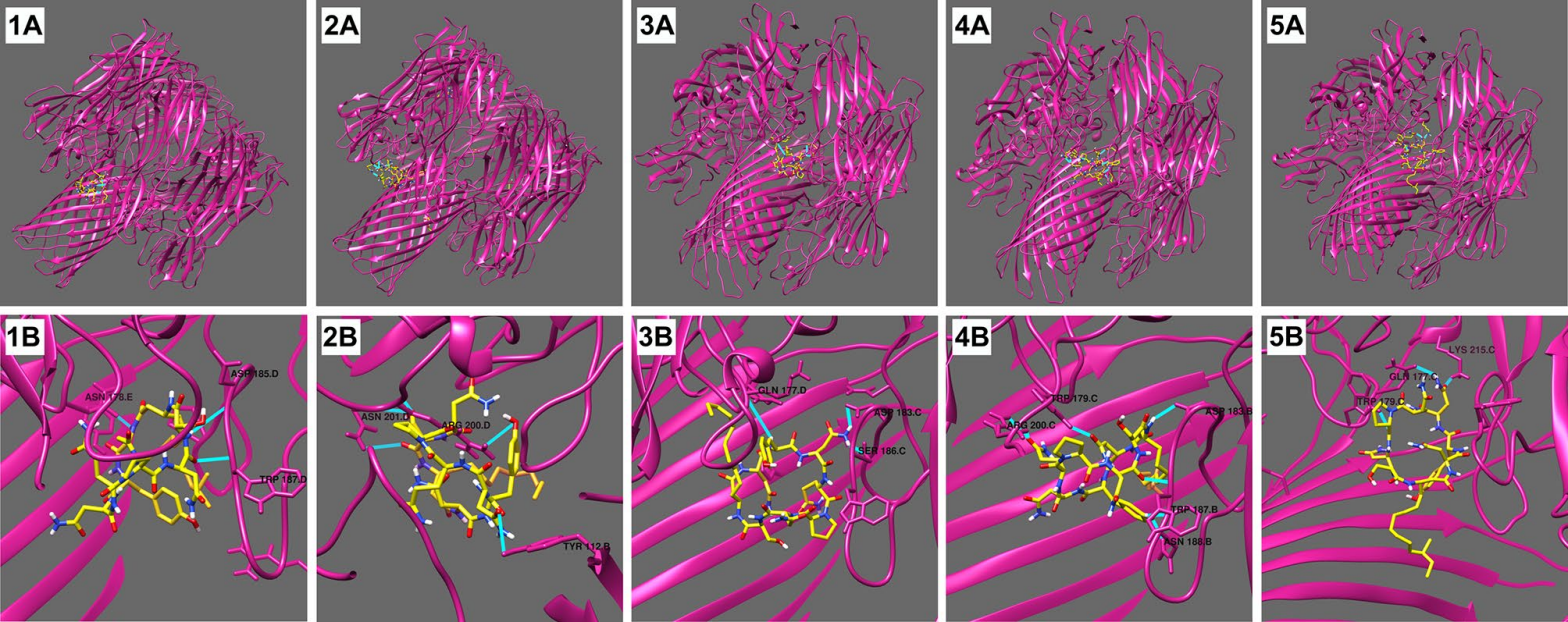
Best molecular models of the interaction between hemolysin and iturins categorized by AutoDock Vina. Top panels (**1A**–**5A**) show the complete heptameric structure of hemolysin interacting with iturins; Bottom panels (**1B**–**5B**) show interactions between the compounds with hydrogen bonds highlighted in light blue. Panels 1, 2, 3, 4, and 5 represent, respectively, the following iturin variants: iturin A, iturin A1, iturin A2, iturin A4, iturin A C-15. The docking analyses were performed using AutoDock Vina v.1.1.2 and images were generated using UCSF Chimera.

**Figure 7:**
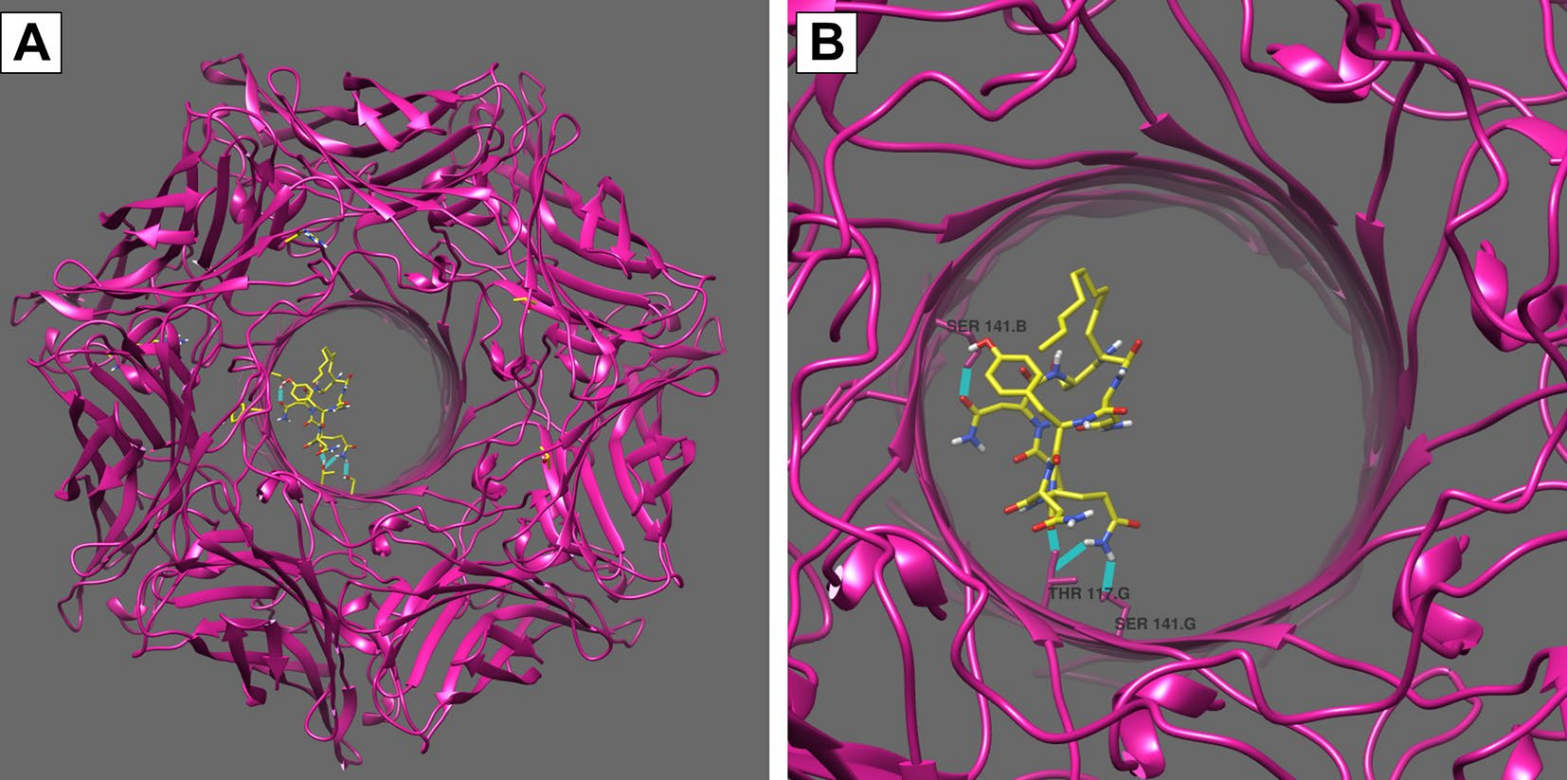
Molecular docking model proposed for hemolysin and iturin A1. Panel (**A**) shows the entire structure while Panel (**B**) highlights the interaction between hemolysin and Iturin A1 through hydrogen bonds is highlighted in light blue. The docking analyses were performed using AutoDock Vina v.1.1.2 and images were generated using UCSF Chimera.

The iturins were predicted to have the capacity to bind to hemolysin, by forming hydrogen bonds with residues from the designed grid box 01 (Supplementary Table 5). The binding affinity of iturins with hemolysin was higher than with ADAM-10, ranging from − 6.6 to − 8.3 kcal/mol according to the best models for each variant. Three to seven hydrogen bonds were established between iturins and hemolysin, most of them with external residues of the heptameric structure (Fig. 6). Iturin A4 showed the best binding affinity and had the highest number of hydrogen bonds with hemolysin, showing its potential as a ligand in vivo. It is also important to highlight the second best model categorized by Autodock Vina for iturin A1 where the ligand bonds inside the pore structure of hemolysin, which could partially explain the blocking of its activity (Fig. 7).

**Figure 8:**
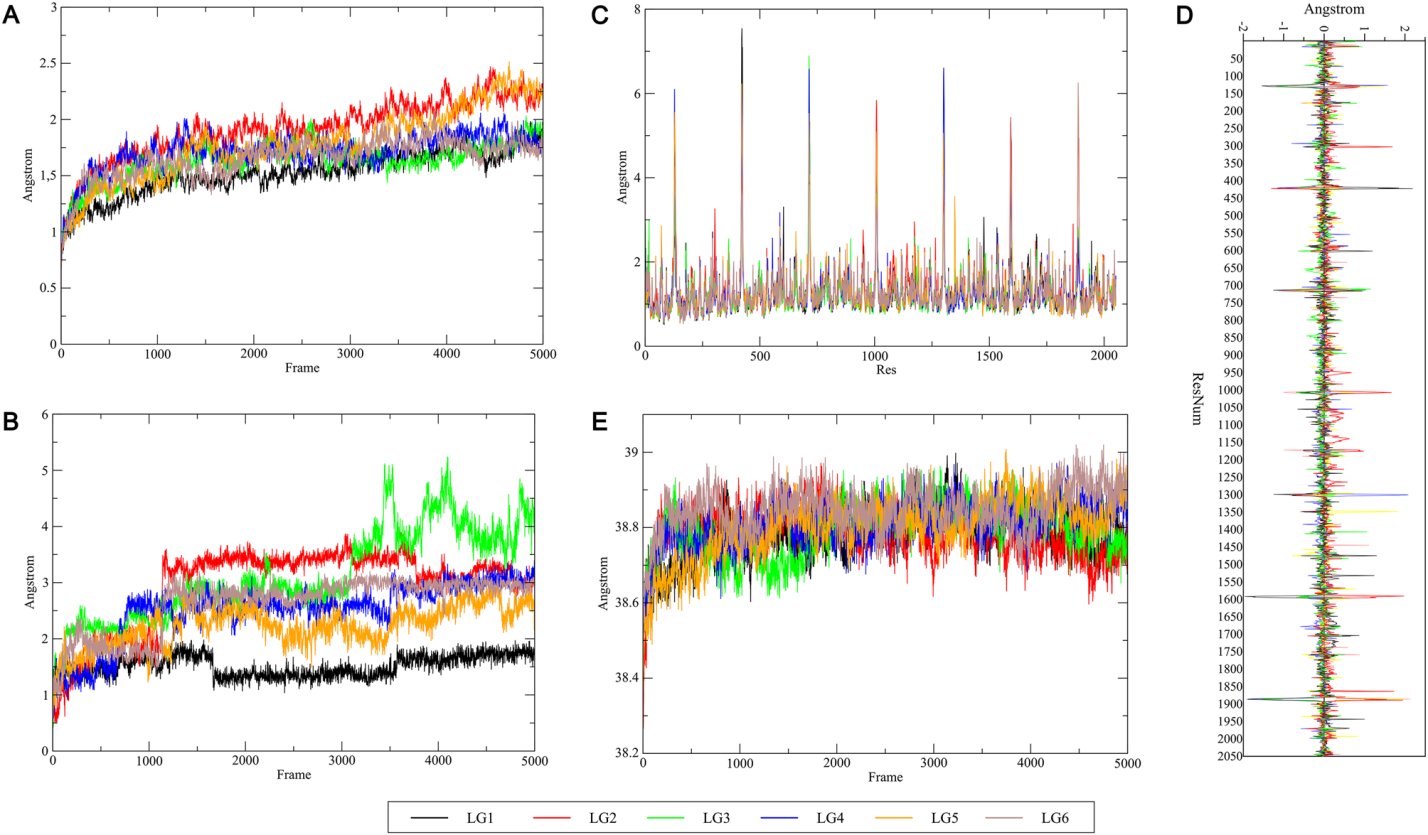
Molecular dynamics results of the complexes composed by the heptameric structure of hemolysin and the iturin variants. In (**A**) is shown the RMSD of the α-carbons present in the protein, and in (**B**) is shown the RMSD of iturins after fitting to the same selection. Panel (**C**) represents the RMSF of protein residues fit to the average structure. Panel (**D**) demonstrates the variation from the mean RMSF of each protein residue in relation to the average structure. Panel (**E**) shows the mass-weighted radius of gyration for the complexes. The complexes were named LG1, LG2, LG3, LG4, LG5 and LG6, corresponding to PubChem CIDs: 9988651, 11062109, 101589794 (poses 1 and 2), 101589795 and 102287549, respectively.

### Molecular dynamics

The root-mean-squared deviation (RMSD) analysis to measure a given frame’s deviation from the structure in the first frame of the trajectory revealed that all systems mostly stabilized in their deviation between frames 500 and 1500, with a mean of 1.69 Å (Fig. 8A). Complexes LG2 and LG5 presented higher deviation, more noticeable from frame 3000 to the end, indicating structural instability. Furthermore, the ligands demonstrated a wider range of conformational variability, with some being relatively stable in deviation (LG1, LG4, LG5 and LG6), while others less so (LG2 and LG3), with a mean of 2.48 Å (Fig. 8B). Nonetheless, at least two conformations were evident in all of them. The discrepancy between deviation at the α-carbon protein and ligand level, especially for LG2 and LG3, is explained by a reduced deviation in the former and notably higher deviation in the latter. The general root mean square fluctuations (RMSF) analysis for protein residues in all 6 systems indicates similar patterns of deviation, with some exceptions dispersed throughout the heptamers chains, with a mean of 1.27 Å (Fig. 8C). However, by analyzing the variation from the mean RMSF, a great variability among the systems is made evident (Fig. 8D). Notably, LG3, LG5 and LG6 showed less variation, with values closer to the mean. Meanwhile, extremes were more common in LG1, LG2 and LG4. Considering important residues for hemolysin activity, the region between residues 179 to 200 (analogous to window 472 to 493 and so on, with an offset of 293 residues between chains), displayed only slight variation, indicating a general stability of these residues along the simulations. In contrast, the nearby windows, represented by residues 102–144, presented greater levels of variation, indicating both the presence of less flexible regions (negative) and more flexible ones (positive).

Rg analysis, linked to the compactness of a complex, demonstrated a marginal increase in value over time, indicating that the systems might become slightly more extended and unstable over time (Fig. 8D). The average Rg value for all systems was 38.80 Å (Fig. 8E). The mean values of RMSD, RMSF and Rg for each system analyzed are summarized in Supplementary Table 6.

Lastly, the analysis of the most prevalent (fraction of frames present $\ge$ 20%) hydrogen bonds (HBs) formed between each respective ligand and hemolysin along each trajectory, revealed the presence of hydrogen bonds with important residues for hemolysin activity (Supplementary Table 7). Remarkably, the residues in the interval 177–201, a buffered region encompassing the important residues for hemolysin binding (179–200), were all present among this HB set: Gln177, Asn178 and Trp187 (3 times), Asp183, Arg200 and Asn201 (2 times) and Trp179, Asp185, Ser186 and Asn188 (only once). Particularly, Asn178, Gln177, Arg200, Trp187 and Asp183 were all seen in more than 40% of the frames. Also noteworthy is a new set of residues, composed of Tyr698, Tyr118, Tyr411, Thr701, Tyr1876 and Tyr405, which also exceed 40% in presence. Relative to ligands, LG5, LG1 and LG6, in that order, held the most of these longevous interactions.

## Discussion

*S. aureus* is an important pathogen for both humans and animals and its resistance to multiple antibiotics has increased the need for therapeutic alternatives to treat infectious diseases caused by this bacterium. As bactericidal therapies have been associated with the development of resistance, therapeutic strategies that affect the ability of bacteria to cause disease without killing the microorganism may be advantageous by delaying the adaptative process^8–10^. In this way, the anti-virulence strategy has emerged as an alternative therapy to treat *S. aureus* infections, in an attempt to control *S. aureus* pathogenicity, while offering a possible long-term effective medicine.

In bovine mastitis, toxin secretion plays a role in the invasion, penetration, and destruction of the mammary gland tissue by *S. aureus*^12^. A study has shown that cows infected with a double *hla* and *hlb* mutant strain were able to eliminate the pathogen and had only mild symptoms compared to severe mastitis caused by the parental strain^45^. Alpha hemolysin is the best-studied hemolysin produced by *S. aureus*, consisting of a small pore-forming cytotoxin secreted as a 33 kDa water-soluble monomer that assembles into a heptamer to form a pore in a wide variety of host cells^46,47^. Several anti-hemolytic compounds have been investigated, including antibodies^48,49^, nanoparticles^50–52^, and natural compounds^53–55^. Some of the synthetic/purified compounds evaluated in these studies were capable, at certain concentrations, to completely abolish the hemolytic activity of *S. aureus*, which agrees with the results reported in the present study. However, most compounds with anti-hemolytic activity described so far are directed toward human pathogens, and none of these candidates evolved to clinical trials. Moreover, here we describe for the first time a crude bacterial extract that can completely inhibit the hemolytic activity of *S. aureus*, which is a cost-attractive option for industrial applications.

In this study, we found that lipopeptides, especially iturins, produced by *B. velezensis* have anti-hemolytic effects against *S. aureus* isolated from bovine mastitis in a dose-dependent manner. Our findings indicate that lipopeptides decrease hemolysin activity rather than its production and that iturins do not interfere with hemolysin oligomerization. However, molecular docking studies predict that iturins are capable of binding to the hemolysin structure in silico, which could potentially explain their effect on hemolysin activity.

Several lipopeptides have demonstrated antimicrobial activity against *S. aureus*, as is the case of the antibiotic daptomycin used to treat severe Gram-positive infections, the first molecule of this class to reach commercial application^24^. Lipopeptides have also demonstrated anti-biofilm activity^56,57^ and anti-quorum sensing effects^17^ against *S. aureus*. Fengycin produced by a probiotic *Bacillus* inhibited the quorum sensing system of *S. aureus* and prevented its colonization in the human intestine^17^. Despite these advances, the current study is the first to demonstrate a direct anti-hemolytic effect of lipopeptides in *S. aureus* without affecting bacterial growth.

Our findings indicate that the expression of genes involved in hemolysin production, including *hla*, *hlb* and *hlg*, increased when *S. aureus* was treated with lipopeptides extracted from *B. velezensis* 87 and *B. velezensis* TR47II. Furthermore, the expression of the genes *agrA* and *agrC*, which positively regulates the expression of toxins^18^, were upregulated in our treatments. However, although the expression of these genes significantly increased in our treatments, it should be noted that only *hlg* had an expressive increase compared to the control. The increase in hemolysin gene expression may be due to a stress response mediated by sigma B, which regulates gene transcription in *S. aureus* during stress potentially increasing *hla* expression^58^. Indeed, in *B. subtilis*, for example, high amounts of surfactin increased the production of stress proteins under the control of alternative sigma factors, such as sigma B and sigma W^59^. Hemolysin-encoded genes (*hlb* and *hlg*) were also upregulated during alcohol-induced stress^60^. The increase in expression of these proteins also does not confirm an increased production or activity of the respective toxins. The *hld* gene, which encodes a delta-toxin, was the only gene downregulated under the conditions evaluated in the current study. Delta toxin is transcribed together with RNAIII, the effector of the agr system^18^. Our results also indicated that the quorum sensing genes of *S. aureus* were upregulated. Therefore, the reduction in the expression of *hld* might be overcome by an increased expression of the quorum sensing genes. In general, these results do not support the observed anti-hemolytic phenotypes, suggesting that lipopeptides could be affecting hemolysin activity rather than its production. Indeed, we verified that the hemolytic activity of *S. aureus* decreased significantly compared to control when *S. aureus* cell-free supernatants were treated with lipopeptides from *Bacillus*, indicating that these molecules inhibit hemolysin activity.

To better understand the mechanism of action of these compounds, we purified the lipopeptides using C18 Solid Phase Extraction and reverse-phase HPLC. Previous studies indicated that due to their different degrees of hydrophobicity, iturins are eluted first in reverse-phase chromatography, followed by fengycins and surfactins^41,43^. Our results demonstrated a higher concentration of fengycin and iturin in *B. velezensis* 87 and *B. velezensis* TR47II extracts, respectively, which explains the higher toxicity of the *B. velezensis* TR47II crude extracts^61^. Surfactins were produced in smaller quantities by both bacteria under the tested conditions. The identification of the fractioned lipopeptides was confirmed by MALDI-TOF mass spectrometry analysis.

Iturin, fengycin, and surfactin are families of lipopeptides with remarkable structural heterogeneity given the diverse peptide sequences, the nature of the peptide cyclization, and the variable length and branching of the fatty acid chain^14^. Iturins are composed of a heptapeptide linked to a beta-amino fatty acid chain of 14–17 carbons in length^15,62^. Iturin variants include iturin A and C, bacillomycin D, F and L, mycosubtilin, mixirin, subtulene A, and mojavensin A^63^. Fengycins are decapeptides linked to a beta-hydroxyl fatty acid chain of 14 to 21 carbon units and are categorized into fengycin A, B, C and Z and plispastatins A and B based on the difference in their sequence and structure^63^. Lastly, surfactins have their heptapeptide linked to a betahydroxy fatty acid chain composed of 12 to 17 carbons^64^. The variants found for the surfactin group were esperin, lichenysin, pumilacidin and surfactin^14^. In this study, the mass spectra results indicate the production of iturin A C14 and C15 by *B. velezensis* 87 and bacillomycin L C14 and C15 by *B. velenzensis* TR47II. Both fengycin C16 and C17 and surfactin C14 and C15 are potentially produced by both strains. Lipopeptides from the same class showed similar effects on *S. aureus* hemolysis, with iturins being the most effective to inhibit hemolytic activity. The observed differences in iturin activity in the hemolysis assays may be explained by the production of different iturin variants by *B. velezensis*. Fengycins showed lower anti-hemolytic activity compared to iturins even at higher concentrations and surfactins were the lipopeptides with the least anti-hemolytic activity. To the best of our knowledge, this is the first report describing the anti-hemolytic activity of lipopeptides, specifically iturins, which prevented hemolysis by *S. aureus* in a range of concentrations. It should also be noted that *Bacillus* spp. crude extracts showed higher anti-hemolytic activity compared to the purified lipopeptides, suggesting synergistic interactions of the compounds released in the cell-free supernatants. This is advantageous from an industrial perspective, since the purification of biomolecules is an expensive process that often limits practical applications.

We also evaluated the mechanism of action of these compounds and found that lipopeptides interfere with the activity of hemolysins. Although the compounds did not act by preventing the oligomerization of hemolysin, molecular docking studies revealed a good binding affinity of iturins with the heptameric structure of hemolysin. The iturins were predicted to bind to the heptameric structure of hemolysin through interactions with external residues, including Trp179, Asp183, Asp185, and Arg200. Arg200 is implicated in membrane binding and cell lysis, while Asp183 and Asp185 are involved in cell lysis^65,66^. Indeed, several studies have demonstrated the ability of anti-virulence compounds to bind to Trp179 and Arg200, showing the importance of these residues for hemolytic activity^30–32^. Another proposed binding model for iturin A1 and hemolysin shows the interaction of the ligand with the protein within the pore structure, which could be a possible mechanism by which iturin blocks the hemolytic activity. Likewise, an anti-hemolytic effect of Isatin-Schiff copper(II) complexes was attributed to the blockage of the channel formed by alpha-hemolysin caused by interactions with the complexes^29^.

We further examined the potential interactions between iturins and hemolysin through molecular dynamics analysis. Despite observing a slight increase in instability of the complexes over time, we noted overall stability in the systems, particularly concerning the critical binding residues numbered 179 to 200. Furthermore, we identified hydrogen bonds between the iturin variants, particularly the lower molecular weight ones like Iturin A, iturin A1, and iturin A2, and hemolysin involving the aforementioned residues across a significant portion of the simulation. This finding underscores the crucial role of this region in stabilizing the complex. Notably, hydrogen bonds with other residues were present in up to 81% of the frames, suggesting they merit deeper investigation. Overall, our analysis indicates that all analyzed iturin variants can bind to hemolysin, offering a partial explanation for the observed anti-hemolytic activity. Furthermore, these results lend support to the hypothesis of synergistic action among the variants, reinforcing the enhanced activity observed in crude extracts containing a mixture of lipopeptides compared to purified compounds..

In summary, in addition to the biological functions already described for iturins such as anti-fungal^67,68^, anti-bacterial^69,70^, and anti-cancer^71,72^, this study reveals, for the first time, that these molecules also have anti-hemolytic potential. This study provides novel insights into the use of iturins/lipopeptides as anti-virulence agents against *S. aureus*. Based on our computational simulations, we suggest that the anti-hemolytic effects of iturins may stem from their direct interaction with hemolysin. However, we acknowledge that additional experiments are necessary to explore whether iturins influence hemolysin binding to cells and the specific mechanisms involved in pore formation. To further validate these findings, in vivo studies are warranted to evaluate the effectiveness of iturins in preventing or treating *S. aureus* infections. Additionally, assessing the cytotoxicity of these compounds can offer insights into their safety profile and therapeutic potential for mastitis treatment.

### Supplementary Information

Supplementary Information.

## Data Availability

All data analyzed and generated in the current study are presented in the manuscript.

## References

[1] Murray CJ (2022). Global burden of bacterial antimicrobial resistance in 2019: A systematic analysis. The Lancet.

[2] Haag AF, Fitzgerald JR, Penadés JR (2019). *Staphylococcus aureus* in animals. Microbiol. Spectr..

[3] Halasa T, Huijps K, Østerås O, Hogeveen H (2007). Economic effects of bovine mastitis and mastitis management: A review. Vet. Q..

[4] Zaatout N, Ayachi A, Kecha M (2020). *Staphylococcus aureus* persistence properties associated with bovine mastitis and alternative therapeutic modalities. J. Appl. Microbiol..

[5] Rainard P (2018). Knowledge gaps and research priorities in *Staphylococcus aureus* mastitis control. Transbound. Emerg. Dis..

[6] Bardiau M, Detilleux J, Farnir F, Mainil JG, Ote I (2014). Associations between properties linked with persistence in a collection of *Staphylococcus aureus* isolates from bovine mastitis. Vet. Microbiol..

[7] Tacconelli E (2018). Discovery, research, and development of new antibiotics: the WHO priority list of antibiotic-resistant bacteria and tuberculosis. Lancet Infect. Dis..

[8] Sully EK (2014). Selective chemical inhibition of agr quorum sensing in *Staphylococcus aureus* promotes host defense with minimal impact on resistance. PLoS Pathog..

[9] Quave CL (2015). *Castanea sativa* (European Chestnut) leaf extracts rich in ursene and oleanene derivatives block *Staphylococcus aureus* virulence and pathogenesis without detectable resistance. PLoS One.

[10] Vale PF (2016). Beyond killing: Can we find new ways to manage infection?. Evol. Med. Public Health.

[11] Fleitas-Martinez O, Cardoso MH, Ribeiro SM, Franco OL (2019). Recent advances in anti-virulence therapeutic strategies with a focus on dismantling bacterial membrane microdomains, toxin neutralization, quorum-sensing interference and biofilm inhibition. Front. Cell. Infect. Microbiol..

[12] Cote-Gravel J, Malouin F (2019). Symposium review: Features of *Staphylococcus aureus* mastitis pathogenesis that guide vaccine development strategies. J. Dairy Sci..

[13] Mayer K (2021). Within-host adaptation of *Staphylococcus aureus* in a bovine mastitis infection is associated with increased cytotoxicity. Int. J. Mol. Sci..

[14] Ongena M, Jacques P (2008). *Bacillus* lipopeptides: Versatile weapons for plant disease biocontrol. Trends Microbiol..

[15] Zhao H (2017). Biological activity of lipopeptides from *Bacillus*. Appl. Microbiol. Biotechnol..

[16] Rivardo F, Turner RJ, Allegrone G, Ceri H, Martinotti MG (2009). Anti-adhesion activity of two biosurfactants produced by *Bacillus* spp. prevents biofilm formation of human bacterial pathogens. Appl. Microbiol. Biotechnol..

[17] Piewngam P (2018). Pathogen elimination by probiotic *Bacillus* via signalling interference. Nature.

[18] Le KY, Otto M (2015). Quorum-sensing regulation in *Staphylococci*—an overview. Front. Microbiol..

[19] Brito M, Brito J, Ribeiro M, Veiga V (1999). Padrão de infecção intramamária em rebanhos leiteiros: Exame de todos os quartos mamários das vacas em lactação. Arq. Bras. Med. Vet. Zootec..

[20] Brito, M. & Brito, J. R. F. *Diagnóstico Microbiológico da Mastite* (Embrapa Gado de Leite Juiz de Fora, 1999).

[21] Le Marechal C (2011). Genome sequences of two *Staphylococcus aureus* ovine strains that induce severe (strain O11) and mild (strain O46) mastitis. J. Bacteriol..

[22] Da Silva ER (2005). Hemolysin production by *Staphylococcus aureus* species isolated from mastitic goat milk in Brazilian dairy herds. Small Rumin. Res..

[23] Tang F (2019). Inhibition of alpha-hemolysin expression by resveratrol attenuates *Staphylococcus aureus* virulence. Microb. Pathog..

[24] Morikawa M, Hirata Y, Imanaka T (2000). A study on the structure-function relationship of lipopeptide biosurfactants. Biochim. Biophys. Acta.

[25] Sharma D (2015). Structural characterization and antimicrobial activity of a biosurfactant obtained from *Bacillus pumilus* DSVP18 grown on potato peels. Jundishapur J. Microbiol..

[26] Untergasser A (2007). Primer3Plus, an enhanced web interface to Primer3. Nucleic Acids Res..

[27] Dong J (2013). Oroxylin A inhibits hemolysis via hindering the self-assembly of alpha-hemolysin heptameric transmembrane pore. PLoS Comput. Biol..

[28] von Hoven G, Meyenburg M, Neukirch C, Siedenschur D, Husmann M (2022). Glutamine 666 renders murine ADAM10 an inefficient *S. aureus* alpha-toxin receptor. BioRxiv.

[29] Melo MC, Teixeira LR, Pol-Fachin L, Rodrigues CGJFML (2016). Inhibition of the hemolytic activity caused by *Staphylococcus aureus* alpha-hemolysin through isatin-Schiff copper (II) complexes. FEMS Microbiol. Lett..

[30] Liu J, Kozhaya L, Torres VJ, Unutmaz D, Lu M (2020). Structure-based discovery of a small-molecule inhibitor of methicillin-resistant *Staphylococcus aureus* virulence. J. Biol. Chem..

[31] Ghoneim MM (2022). Proposed mechanism for emodin as agent for methicillin resistant *Staphylococcus aureus*. In vitro testing and in silico study. Curr. Issues. Mol. Biol..

[32] Wan S-J (2022). Two novel phenylpropanoid trimers from ligusticum chuanxiong hort with inhibitory activities on alpha-hemolysin secreted by *Staphylococcus aureus*. Front. Chem..

[33] Morris GM (2009). AutoDock4 and AutoDockTools4: receptor flexibility. J. Comput. Chem..

[34] Csizmadia, P. MarvinSketch and MarvinView: Molecule applets for the World Wide Web. *The 3rd International Electronic Conference on Synthetic Organic Chemistry session Information and Compound Archives Management and Internet Application* (1999).

[35] Trott O, Olson AJ (2010). AutoDock Vina: Improving the speed and accuracy of docking with a new scoring function, efficient optimization, and multithreading. J. Comput. Chem..

[36] Pettersen EF (2004). UCSF Chimera—a visualization system for exploratory research and analysis. J. Comput. Chem..

[37] Phillips JC (2020). Scalable molecular dynamics on CPU and GPU architectures with NAMD. J. Chem. Phys..

[38] Case DA (2023). Amber tools. J. Chem. Inf. Model..

[39] Beadell B, Nehra S, Gusenov E, Huse H, Wong-Beringer A (2023). Machine learning with alpha toxin phenotype to predict clinical outcome in patients with *Staphylococcus aureus* bloodstream infection. Toxins Basel.

[40] Pathak KV, Keharia H (2014). Identification of surfactins and iturins produced by potent fungal antagonist, *Bacillus subtilis* K1 isolated from aerial roots of banyan (*Ficus benghalensis*) tree using mass spectrometry. 3 Biotech.

[41] Yang H, Li X, Li X, Yu H, Shen Z (2015). Identification of lipopeptide isoforms by MALDI-TOF-MS/MS based on the simultaneous purification of iturin, fengycin, and surfactin by RP-HPLC. Anal. Bioanal. Chem..

[42] Li XY (2012). ESI LC-MS and MS/MS characterization of antifungal cyclic lipopeptides produced by Bacillus subtilis XF-1. J. Mol. Microbiol. Biotechnol..

[43] Kim PI, Ryu J, Kim YH, Chi YT (2010). Production of biosurfactant lipopeptides Iturin A, fengycin and surfactin A from *Bacillus subtilis* CMB32 for control of *Colletotrichum gloeosporioides*. J. Microbiol. Biotechnol..

[44] Dunlap CA, Bowman MJ, Rooney AP (2019). Iturinic lipopeptide diversity in the *Bacillus subtilis* species group—important antifungals for plant disease biocontrol applications. Front. Microbiol..

[45] Kenny K, Bastida FD, Norcross NL (1992). Secretion of alpha-hemolysin by bovine mammary isolates of *Staphylococcus aureus*. Can. J. Vet. Res..

[46] Berube BJ, Wardenburg JB (2013). *Staphylococcus aureus* α-toxin: Nearly a century of intrigue. Toxins (Basel).

[47] Divyakolu S, Chikkala R, Ratnakar KS, Sritharan V (2019). Hemolysins of *Staphylococcus aureus*—an update on their biology, role in pathogenesis and as targets for anti-virulence therapy. Adv. Infect. Dis..

[48] Foletti D (2013). Mechanism of action and in vivo efficacy of a human-derived antibody against *Staphylococcus aureus* alpha-hemolysin. J. Mol. Biol..

[49] Francois B (2018). Safety and tolerability of a single administration of AR-301, a human monoclonal antibody, in ICU patients with severe pneumonia caused by *Staphylococcus aureus*: First-in-human trial. Intensive Care Med..

[50] Henry BD (2015). Engineered liposomes sequester bacterial exotoxins and protect from severe invasive infections in mice. Nat. Biotechnol..

[51] Chen Y (2018). Broad-spectrum neutralization of pore-forming toxins with human erythrocyte membrane-coated nanosponges. Adv. Healthc. Mater..

[52] Keller MD (2020). Decoy exosomes provide protection against bacterial toxins. Nature.

[53] Karginov VA (2007). Inhibition of *S. aureus* alpha-hemolysin and *B. anthracis* lethal toxin by beta-cyclodextrin derivatives. Bioorg. Med. Chem..

[54] Dong J (2013). Apigenin alleviates the symptoms of *Staphylococcus aureus* pneumonia by inhibiting the production of alpha-hemolysin. FEMS Microbiol. Lett..

[55] Wang J (2015). Morin hydrate attenuates *Staphylococcus aureus* virulence by inhibiting the self-assembly of alpha-hemolysin. J. Appl. Microbiol..

[56] De-Zoysa GH, Wang K, Lu J, Hemar Y, Sarojini V (2020). Covalently immobilized battacin lipopeptide gels with activity against bacterial biofilms. Molecules.

[57] Quinn GA, Maloy AP, McClean S, Carney B, Slater JW (2012). Lipopeptide biosurfactants from *Paenibacillus* polymyxa inhibit single and mixed species biofilms. Biofouling.

[58] Chen HY (2011). Vancomycin activates sigma(B) in vancomycin-resistant *Staphylococcus aureus* resulting in the enhancement of cytotoxicity. PLoS One.

[59] Lilge L (2022). Surfactin shows relatively low antimicrobial activity against *Bacillus subtilis* and other bacterial model organisms in the absence of synergistic metabolites. Microorganisms.

[60] Korem M, Gov Y, Rosenberg M (2010). Global gene expression in *Staphylococcus aureus* following exposure to alcohol. Microb. Pathog..

[61] Vanittanakom N, Loeffler W, Koch U, Jung G (1986). Fengycin-a novel antifungal lipopeptide antibiotic produced by *Bacillus subtilis* F-29-3. J. Antibiot..

[62] Raaijmakers JM, De Bruijn I, Nybroe O, Ongena M (2010). Natural functions of lipopeptides from *Bacillus* and *Pseudomonas*: More than surfactants and antibiotics. FEMS Microbiol. Rev..

[63] Cochrane SA, Vederas JC (2016). Lipopeptides from *Bacillus* and *Paenibacillus* spp.: A gold mine of antibiotic candidates. Med. Res. Rev..

[64] Theatre A (2021). The surfactin-like lipopeptides from *Bacillus* spp.: Natural biodiversity and synthetic biology for a broader application range. Front. Bioeng. Biotechnol..

[65] Gouaux E (1998). α-Hemolysin from *Staphylococcus aureus*: An archetype of β-barrel, channel-forming toxins. J. Struct. Biol..

[66] Walker B, Bayley H (1995). Key residues for membrane binding, oligomerization, and pore forming activity of staphylococcal alpha-hemolysin identified by cysteine scanning mutagenesis and targeted chemical modification. J. Biol. Chem..

[67] Cho KM (2009). Iturin produced by *Bacillus pumilus* HY1 from Korean soybean sauce (kanjang) inhibits growth of aflatoxin producing fungi. Food Control.

[68] Han Q (2015). The bacterial lipopeptide iturins induce *Verticillium dahliae* cell death by affecting fungal signalling pathways and mediate plant defence responses involved in pathogen-associated molecular pattern-triggered immunity. Environ. Microbiol..

[69] Asaka O, Shoda M (1996). Biocontrol of *Rhizoctonia solani* damping-off of tomato with *Bacillus subtilis* RB14. Appl. Environ. Microbiol..

[70] Leclère V (2005). Mycosubtilin overproduction by *Bacillus subtilis* BBG100 enhances the organism's antagonistic and biocontrol activities. Appl. Environ. Microbiol..

[71] Dey G (2015). Marine lipopeptide Iturin A inhibits Akt mediated GSK3beta and FoxO3a signaling and triggers apoptosis in breast cancer. Sci. Rep..

[72] Dey G (2017). Therapeutic implication of ‘Iturin A’for targeting MD-2/TLR4 complex to overcome angiogenesis and invasion. Cell Signal..

