# Supplementary material for: Bacillus velezensis iturins inhibit the hemolytic activity of Staphylococcus aureus
Source: Sci Rep. 2024 Apr 24;14:9469. doi: 10.1038/s41598-024-58973-0 (PMC11043418; doi:10.1038/s41598-024-58973-0)
Supplement: Supplementary file 1 — Supplementary Information. [file 41598_2024_58973_MOESM1_ESM.docx]

**SUPPLEMENTARY FIGURES**

**
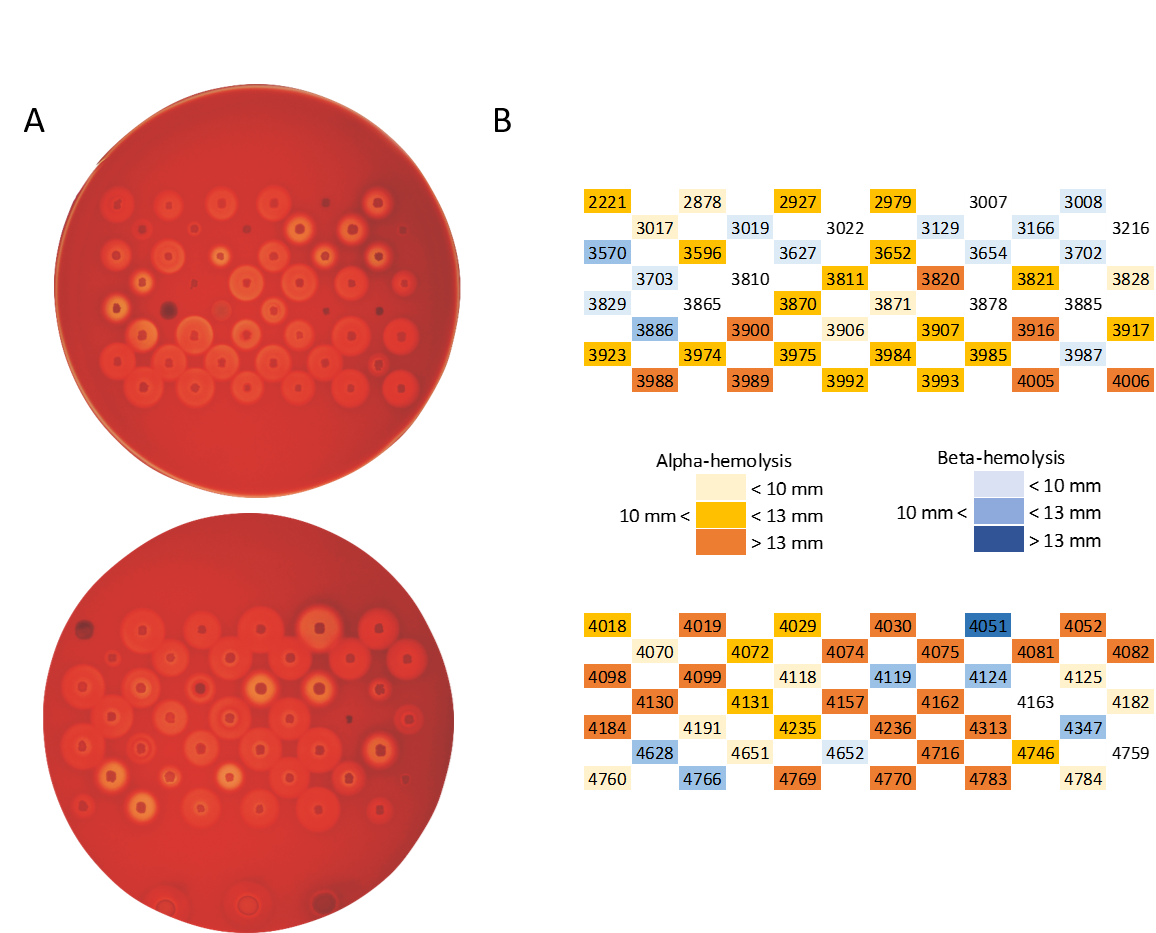
**

**Supplementary Fig. 1.** Hemolytic activity of *S. aureus* isolated from cows with mastitis in sheep blood agar. A) hemolytic activity of the isolates after incubation at 37 °C for 24 h and then at 4 °C overnight. B) identification of the cultures distributed in the sheep blood agar categorized by the type of hemolysis (alpha or beta) and the size of the hemolytic zone.


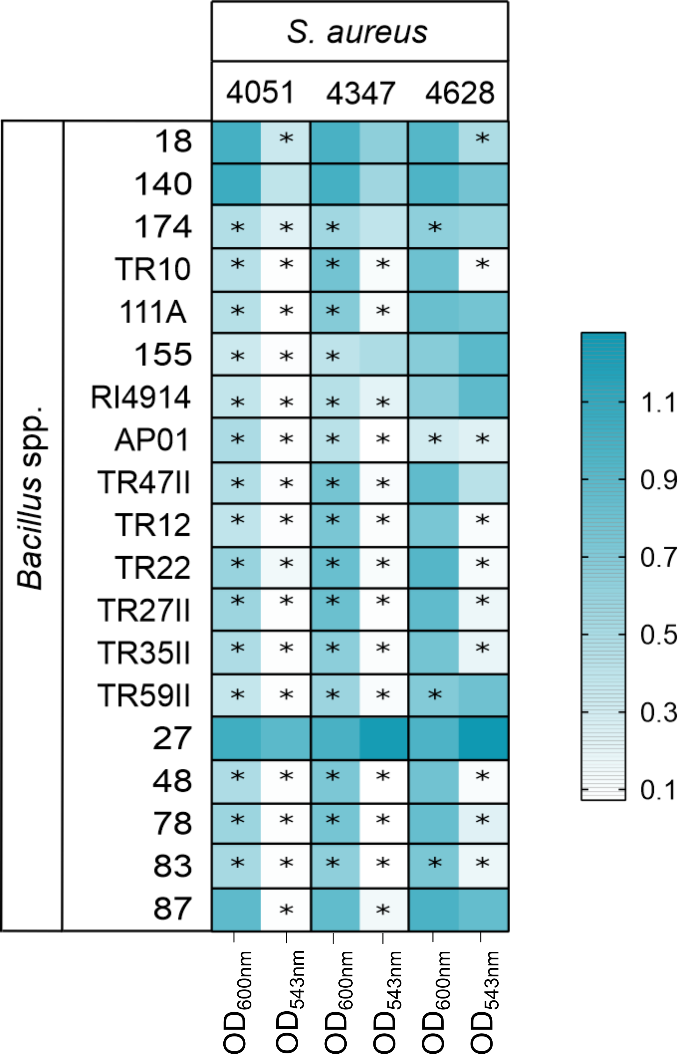


**Supplementary Fig. 2.** Heatmap showing the effects of *Bacillus* supernatants on the growth (OD_600nm_) and hemolytic activity of *S. aureus* (OD_543nm_). *Bacillus* strains are shown on the left side of the figure and *S. aureus* strains are listed on the top. Only *Bacillus* supernatants that did not show hemolytic activity (toxic effect) were included in this analysis. The OD of the controls (without treatment) was normalized to 1.0. Asterisks show a significant difference (p < 0.05) in hemolytic activity between the treated cultures and the control assessed by the Mann-Whitney test.
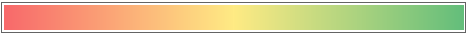


17% 519%


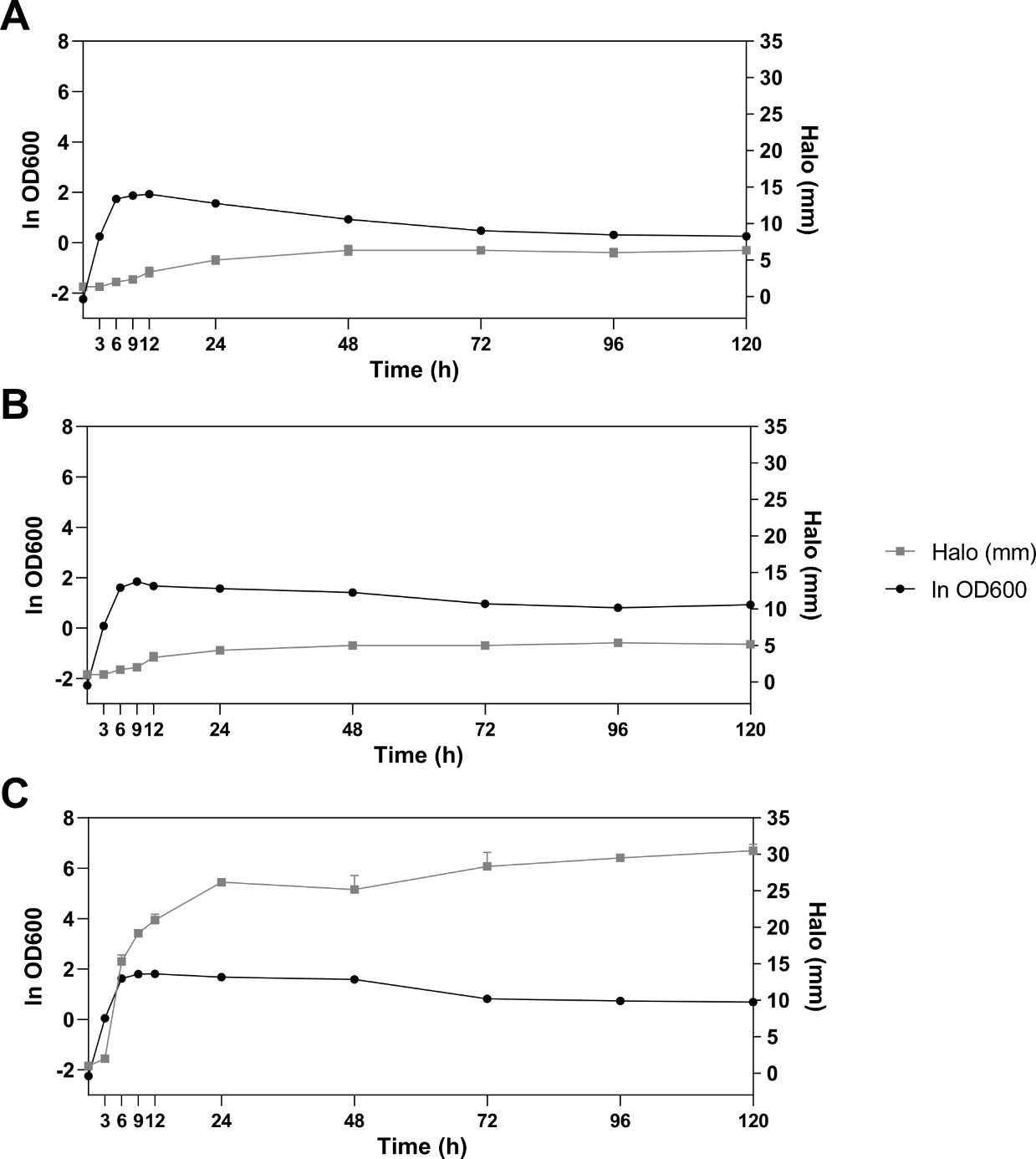


**Supplementary Fig. 3.** Growth curve and production of lipopeptides by *Bacillus* sp. 18 (A), *Bacillus velezensis* 87 (B), and *Bacillus velezensis* TR47II (C). Lipopeptide production was estimated using the crude oil displacement assay and expressed by halo diameter (square symbols). Bacterial growth (OD600nm) is represented by a line connecting filled circles. The experiment was performed using three biological replicates. Bars indicate the standard error of the mean.


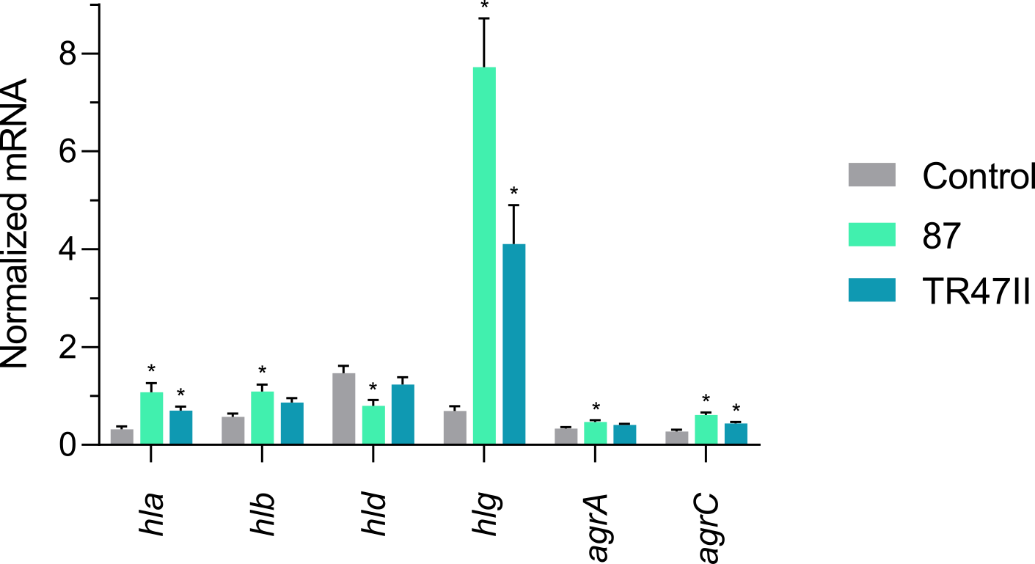


**Supplementary Fig. 4.** Effect of lipopeptide extracts on the expression of genes involved in the synthesis of hemolysins by *S. aureus* O11. *B. velezensis* 87 and *B. velezensis* TR47II are represented by green and blue bars, respectively, and the control is shown in dark gray. Error bars indicate the standard error of the mean. Asterisks represent a significant difference between treatments and the control by the Mann-Whitney test at a 95% confidence level.


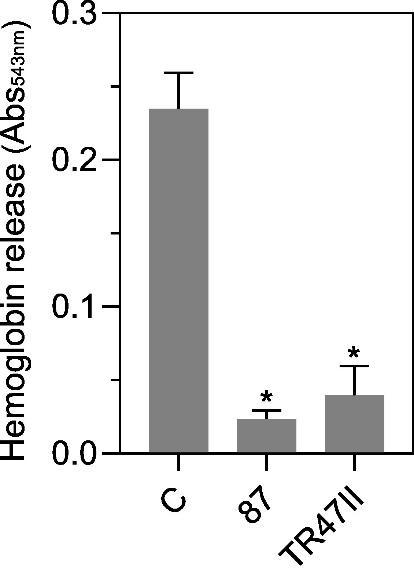


**Supplementary Fig. 5.** Effect of lipopeptides on S. aureus hemolysin activity. The release of hemoglobin by S. aureus O11 was measured after supernatants were treated with lipopeptides from B. velezensis 87 and B. velezensis TR47II at 500 µg/mL and 125 µg/mL, respectively. C represents the hemolytic activity of S. aureus O11 supernatant in the absence of treatments (Control). Asterisk means a significant difference between the treatments and the control evaluated using Mann Whitney t-test.


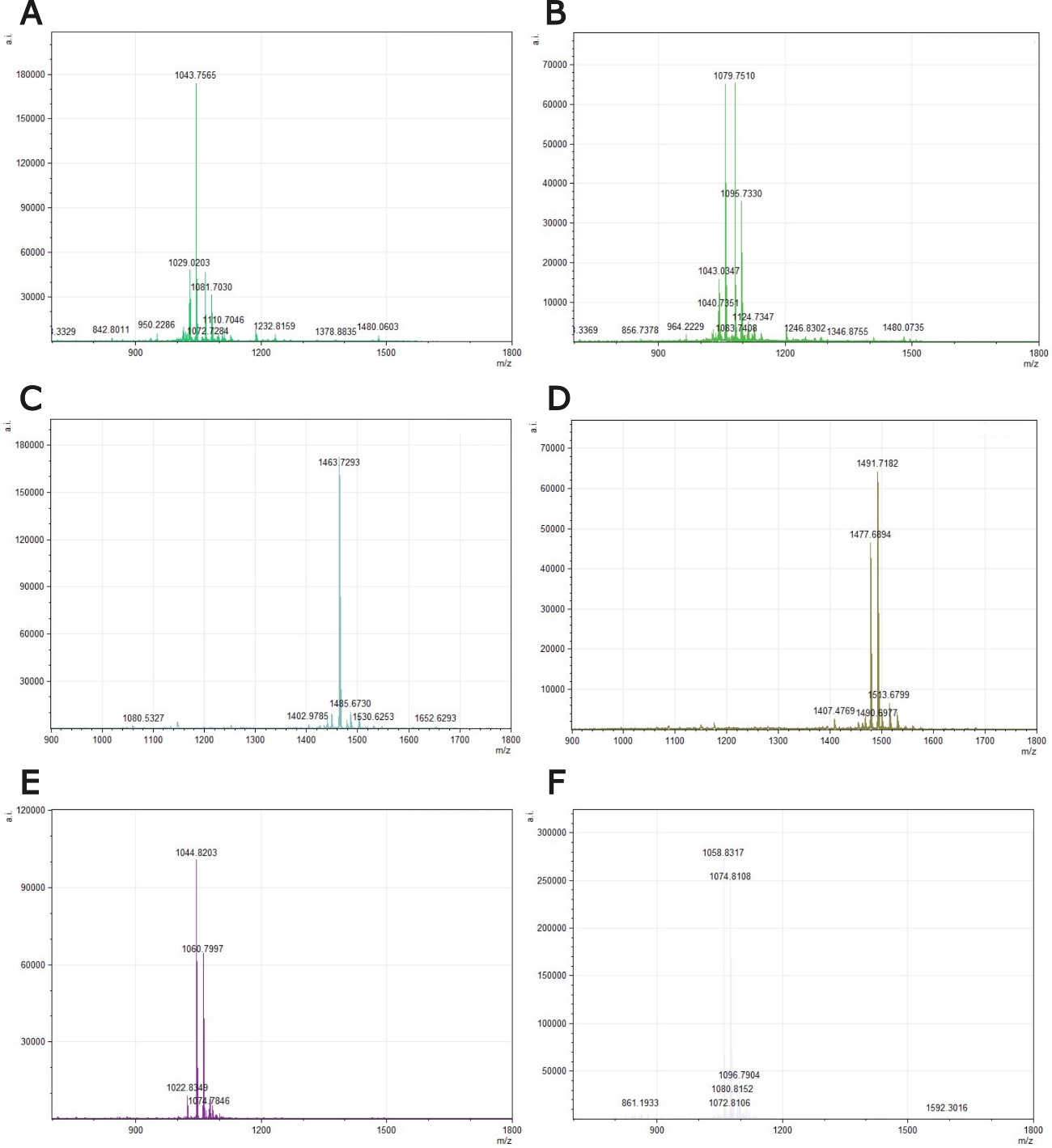


**Supplementary Fig. 6.** MALDI-TOF mass spectrometry of lipopeptides from *Bacillus velezensis* 87 purified by RP-HPLC. A and B, represent, respectively, fractions 23 and 26; C and D represent fractions 38 and 40, and E and F represent fractions 71 and 75.


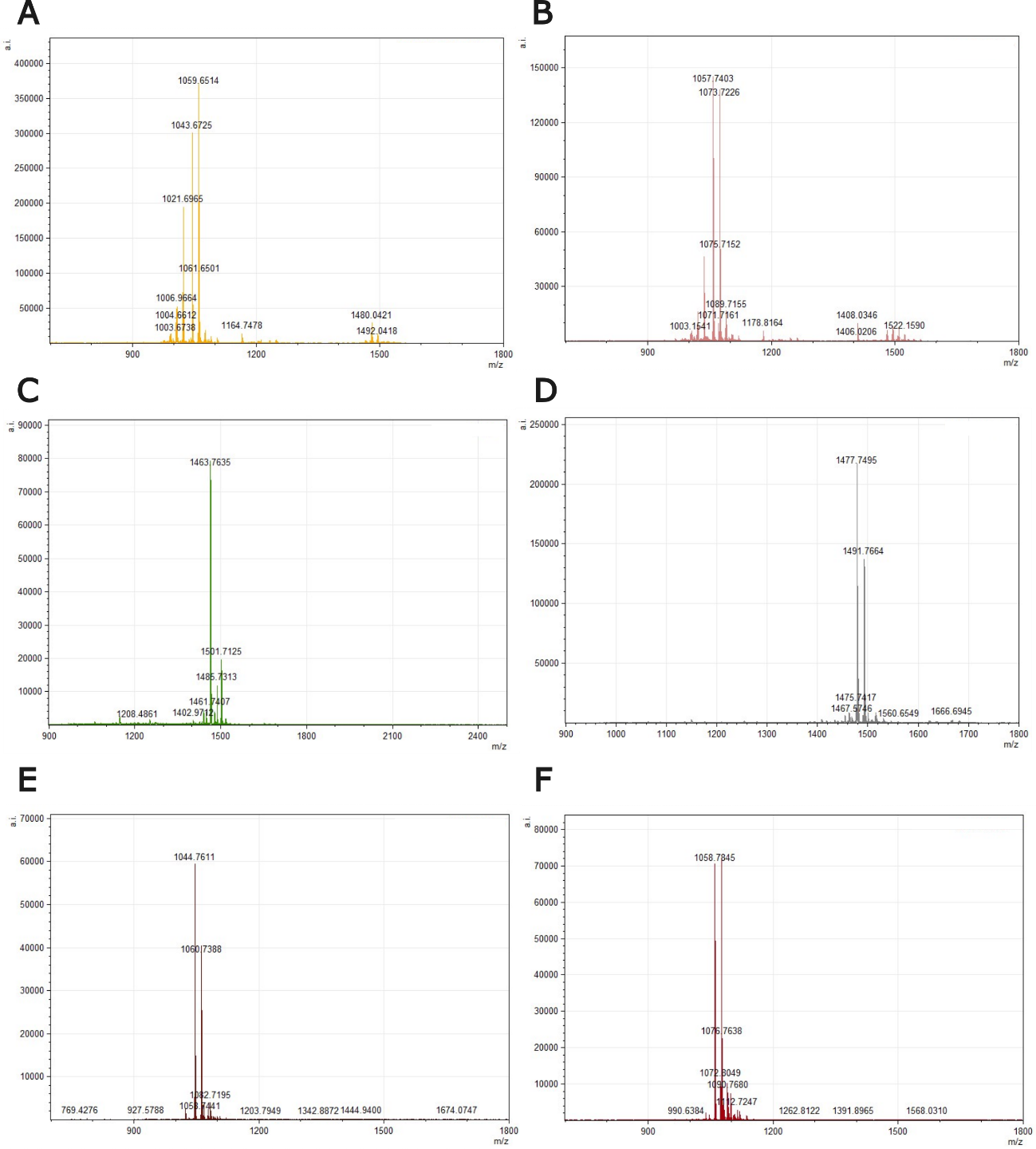


**Supplementary Fig. 7.** MALDI-TOF mass spectrometry of lipopeptides from *Bacillus velezensis* TR47II purified by RP-HPLC. A and B, represent, respectively, fractions 24 and 27; C and D represent fractions 38 and 41, and E and F represent fractions 81 and 84.


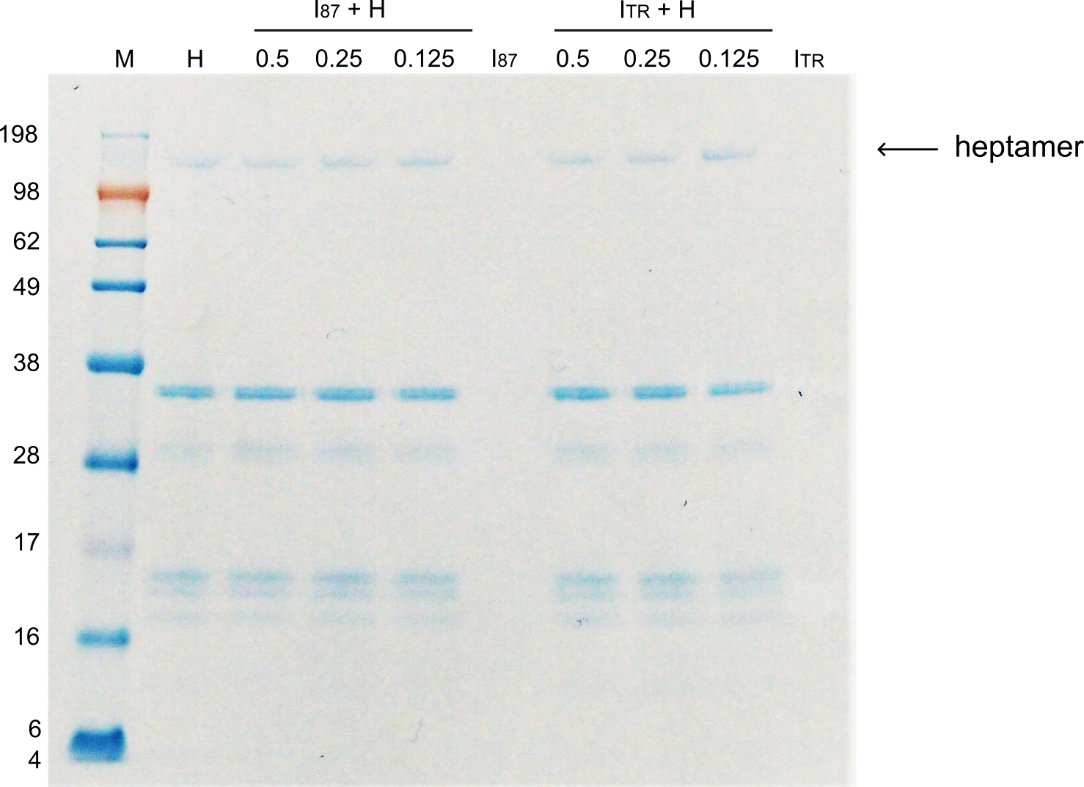


**Supplementary Fig. 8.** Oligomerization assay of α-hemolysin in the presence of iturins. Iturins from *B. velezensis* 87 and *B. velezensis* TR47II were tested at different concentrations. Hemolysin (H) alone was used as a positive control and iturins from both *Bacillus* species were used as a negative control (I_87_ and I_TR_). SeeBlue Plus2 Pre-stained was used as a protein standard (M). The experiment was done using a Nu-Page gel stained with EZBlue.

**SUPPLEMENTARY TABLES**

**Supplementary Table 1.** *Bacillus* strains used in this study

| Bacteria | Method of identification | Source | State/Country | Reference (DOI) |
| --- | --- | --- | --- | --- |
| *B. velezensis* 48 | rRNA 16S and rpoB sequencing | Potato field | MG/Brazil | - |
| *B. velezensis* 78 | rRNA 16S and rpoB sequencing | Mango roots | MG/Brazil | - |
| *B. velezensis* 83 | rRNA 16S and rpoB sequencing | Mango roots | MG/Brazil | - |
| *B. velezensis* 87 | rRNA 16S and rpoB sequencing | Washing of coffee planting soil | MG/Brazil | - |
| *B. cereus/thuringiensis* 32 | rRNA 16S and rpoB sequencing | Forest of the coffee nursery | MG/Brazil | - |
| *B. cereus/thuringiensis* 55 | rRNA 16S and rpoB sequencing | Potato field | MG/Brazil | - |
| *B. cereus/thuringiensis* 90 | rRNA 16S and rpoB sequencing | Washing of coffee planting soil | MG/Brazil | - |
| *B. cereus/thuringiensis* 94 | rRNA 16S and rpoB sequencing | Leaf litter of the coffee nursery | MG/Brazil | - |
| *B. toyonensis* 21 | rRNA 16S and rpoB sequencing | Forest of the coffee nursery | MG/Brazil | - |
| *B. toyonensis* 86 | rRNA 16S and rpoB sequencing | Washing of coffee planting soil | MG/Brazil | - |
| *B. altitudinis* 27 | rRNA 16S and rpoB sequencing | Forest of the coffee nursery | MG/Brazil | - |
| *Bacillus cereus* 12 | Fatty acid methyl esters (FAME) analysis | Bovine mastitis | MT/Brazil | - |
| *Bacillus cereus* 13 | Fatty acid methyl esters (FAME) analysis | Bovine mastitis | MT/Brazil | - |
| *Bacillus* *cereus/thuringiensis* 14 | Fatty acid methyl esters (FAME) analysis | Bovine mastitis | MT/Brazil | - |
| *B. subtilis* LBBMA RI4914 | Fatty acid methyl esters (FAME) analysis | Production water of oil exploration field | ES/Brazil | [10.1016/j.fuel.2016.04.080](https://doi.org/10.1016/j.fuel.2016.04.080) |
| *B. subtilis* LBBMA 111A | Fatty acid methyl esters (FAME) analysis | Mangrove region contaminated with oil | RJ/Brazil | - |
| *B. subtilis* LBBMA AP01 | Fatty acid methyl esters (FAME) analysis | Contamination of an agar plate used for isolation of phytopathogenic fungi | MG/Brazil | - |
| *B. velezensis* TR47II | rRNA 16S sequencing and fatty acid methyl esters (FAME) analysis | Soil | ES/Brazil | 10.1007/s00792-015-0740-7 |
| *B. subtilis* LBBMA 155 | Fatty acid methyl esters (FAME) analysis | Mangrove region contaminated with oil | RJ/Brazil | - |
| *B. subtilis* TR10 | rRNA 16S sequencing and fatty acid methyl esters (FAME) analysis | Soil | ES/Brazil | 10.1007/s00792-015-0740-7 |
| *B. subtilis* TR12 | rRNA 16S sequencing and fatty acid methyl esters (FAME) analysis | Soil | ES/Brazil | 10.1007/s00792-015-0740-7 |
| *B. subtilis* TR22 | rRNA 16S sequencing and fatty acid methyl esters (FAME) analysis | Soil | ES/Brazil | 10.1007/s00792-015-0740-7 |
| *B. subtilis* TR27II | rRNA 16S sequencing and fatty acid methyl esters (FAME) analysis | Soil | ES/Brazil | 10.1007/s00792-015-0740-7 |
| *B. subtilis* TR35II | rRNA 16S sequencing and fatty acid methyl esters (FAME) analysis | Soil | ES/Brazil | 10.1007/s00792-015-0740-7 |
| *B. subtilis* TR59II | rRNA 16S sequencing and fatty acid methyl esters (FAME) analysis | Soil | ES/Brazil | 10.1007/s00792-015-0740-7 |
| *Bacillus* sp. 18 | rRNA 16S sequencing | *Hevea brasiliensis* stalk | AM/Brazil | - |
| *Bacillus* sp. 93 | rRNA 16S sequencing | *Hevea brasiliensis* leaf | AM/Brazil | - |
| *Bacillus* sp. 140 | rRNA 16S sequencing | *Hevea brasiliensis* leaf | AM/Brazil | - |
| *Bacillus* sp. 174 | rRNA 16S sequencing | *Hevea brasiliensis* root | AM/Brazil | - |
| *Bacillus* sp. 201 | rRNA 16S sequencing | *Hevea brasiliensis* root | AC/Brazil | - |
| *Bacillus* sp. 204 | rRNA 16S sequencing | *Hevea brasiliensis* root | AC/Brazil | - |
| *Bacillus* sp. 210 | rRNA 16S sequencing | *Hevea brasiliensis* root | AC/Brazil | - |
| *Bacillus* sp. 221 | rRNA 16S sequencing | *Hevea brasiliensis* leaf | AM/Brazil | - |

**Supplementary Table 2.** Target genes and corresponding primer sequences used in the RT-PCR assay

| **Gene** | **Direction** | **Sequence** | **Product length (bp)** |
| --- | --- | --- | --- |
| *agrA* | F | 5'- CGT GGC AGT AAT TCA GTG TAT G -3' | 83 |
|  | R | 5'- TGG GCA ATG AGT CTG TGA GA -3' |  |
| *agrC* | F | 5'- GAA ATA CCA GAT GAA GTA ACT CGC A -3' | 127 |
|  | R | 5'- ATG CAA CTC GAA TGA TAG GAT C -3' |  |
| *hla* | F | 5'- AGAGATTCTTGGAACCCGGTATATG -3' | 146 |
|  | R | 5'- ATAACTGTAGCGAAGTCTGGTGAA -3' |  |
| *hlb* | F | 5'- GGT TGT GGA TTC GAT AAT GAT AGC -3' | 131 |
|  | R | 5'- CGA TCA TGT CCA GCA CCA -3' |  |
| *hld* | F | 5'- GGA AGG AGT GAT TTC AAT GGC A -3' | 80 |
|  | R | 5'- TGT TCA CTG TGT CGA TAA TCC A -3' |  |
| *hlgC* | F | 5'- CCAATCAGCCCCATCACTCGGT -3' | 130 |
|  | R | 5'- CGCTTTGACGCCCCATAAAACACT -3' |  |

**Supplementary Table 3.** Iturin variants obtained from PubChem

| Variant | Formula | Molecular weight | PubChem CID |
| --- | --- | --- | --- |
| Iturin A | [C_48_H_74_N_12_O_14_](https://pubchem.ncbi.nlm.nih.gov/#query=C48H74N12O14) | 1,043.20 g/mol | 102287549 |
| Iturin A1 | [C_47_H_72_N_12_O_14_](https://pubchem.ncbi.nlm.nih.gov/#query=C47H72N12O14) | 1,029.09 g/mol | 101589794 |
| Iturin A2 | [C_48_H_74_N_12_O_14_](https://pubchem.ncbi.nlm.nih.gov/#query=C48H74N12O14) | 1,043.20 g/mol | 9988651 |
| Iturin A4 | [C_49_H_76_N_12_O_14_](https://pubchem.ncbi.nlm.nih.gov/#query=C49H76N12O14) | 1,057.20 g/mol | 11062109 |
| Iturin A C-15 | [C_49_H_76_N_12_O_14_](https://pubchem.ncbi.nlm.nih.gov/#query=C49H76N12O14) | 1,057.20 g/mol | 101589795 |

**Supplementary Table 4.** Best model result of the molecular docking between ADAM-10 and iturin variants performed by AutoDock Vina

| PubChem CID | Binding Affinity (kcal/mol) | Num. of Hbonds | Residues |
| --- | --- | --- | --- |
| CID_9988651 | -2,7 | 3 | 2x Glu665 Asn669 |
| CID_11062109 | -2,7 | 3 | Ser663 Asn669 Glu 665 |
| CID_101589794 | -2,7 | 3 | 2x Glu665 Glu668 |
| CID_101589795 | -2,9 | 4 | Ser663 Glu665 Glu668 Asn669 |
| CID_102287549 | -2,8 | 4 | Ser663  2x Glu665 Asn669 |

**Supplementary Table 5.** Results of the molecular docking between hemolysin and iturins variants performed by AutoDock Vina

|  | Model 01 | | | Model 02 | | | Model 03 | | | Model 04 | | | | Model 05 | | | |
| --- | --- | --- | --- | --- | --- | --- | --- | --- | --- | --- | --- | --- | --- | --- | --- | --- | --- |
| PubChem CID | **Binding Affinity (kcal/mol)** | **Num. of Hbonds** | **Residues** | **Binding Affinity (kcal/mol)** | **Num. of Hbonds** | **Residues** | **Binding Affinity (kcal/mol)** | **Num. of Hbonds** | **Residues** | **Binding Affinity (kcal/mol)** | **Num. of Hbonds** | **Residues** | **Binding Affinity (kcal/mol)** | | **Num. of Hbonds** | **Residues** |  |
| CID_9988651 | -6,6 | 3 | Gln177 Asp183  Ser 186 | -6,6 | 3 | Gln177 Asp183 Trp187 | -6,4 | 4 | Gln177 Asp183 Asn173 Trp187 | -6,4 | 3 | Gln177 His144 Lys198 | -6,3 | | 4 | Thr155 Asp227 Ser225 Arg104 |  |
| CID_11062109 | -8,3 | 7 | 2x Arg200 Asp183  Trp 179  2x Trp187 Asn188 | -7,4 | 2 | Thr155 Asp227 | -7,3 | 2 | Asn188 Asp185 | -7,3 | 2 | Gln177 Asn173 | -7,3 | | 2 | Trp187 Tyr118 |  |
| CID_101589794 | -7,1 | 5 | Tyr 112  2x Arg200  2x Asn201 | -6,9 | 4 | 2x Thr117 2x Ser141 | -6,8 | 1 | Thr155 | -6,6 | 1 | Thr155 | -6,5 | | 2 | 2x Arg104 |  |
| CID_101589795 | -7,7 | 3 | Lys 215  Gln 177 Trp 179 | -7,6 | 3 | Asn 178 Arg 200 Tyr 182 | -7,5 | 4 | Trp 187 Ser 186 Gln 177 Trp 179 | -7,5 | 2 | Asp227 Thr155 | -7,4 | | 4 | Trp 187  His 144  Gln 177 Asn 201 |  |
| CID_102287549 | -7,1 | 3 | Asn 178 Trp 187 Asp 185 | -6,6 | 3 | Trp 187 Asn 178 Tyr 112 | -6,6 | 3 | Lys 198 Arg 200 His 144 | -6,5 | 2 | Trp 187 Pro 181 | -6,4 | | 5 | Lys 215  Pro 181  Gly 180  Tyr 112  Hys 144 |  |

**Supplementary Table 6.** Summary of the mean values for each system studied in the molecular dynamic analysis

| **Parameters (Å)** | **LG1** | **LG2** | **LG3** | **LG4** | **LG5** | **LG6** |
| --- | --- | --- | --- | --- | --- | --- |
| Protein RMSD | 1.51821 | 1.90441 | 1.61555 | 1.69433 | 1.77311 | 1.64181 |
| Ligand RMSD | 1.50420 | 2.91737 | 3.11555 | 2.51120 | 2.21709 | 2.60924 |
| RMSF | 1.25481 | 1.34729 | 1.23608 | 1.24438 | 1.28151 | 1.27767 |
| Rg | 38.77750 | 38.78210 | 38.789300 | 38.80610 | 38.79940 | 38.84650 |

**Supplementary Table 7.** Most frequent hydrogen bonds along the trajectory of each system. A 20% fraction was applied as a cutoff to select the most prevalent interactions

| **System** | **Acceptor*** | **DonorH*** | **Donor** | **Frames** | **Fraction** | **AvgDist** | **AvgAng** |
| --- | --- | --- | --- | --- | --- | --- | --- |
| LG6 | LG6_2052@O7 | TYR_698@HH | TYR_698@OH | 4088 | 0.8176 | 2.7621 | 160.2202 |
| LG1 | LG1_2052@O9 | **ASN_1057@HD22** | ASN_1057@ND2 | 3775 | 0.7550 | 2.8368 | 160.4433 |
| LG1 | **GLN_1056@O** | LG1_2052@H6 | LG1_2052@N7 | 3565 | 0.7130 | 2.7836 | 148.0039 |
| LG1 | LG1_2052@O5 | TYR_118@HH | TYR_118@OH | 3245 | 0.6490 | 2.7567 | 163.8131 |
| LG3 | LG3_2052@O3 | **ARG_1079@H** | ARG_1079@N | 3222 | 0.6444 | 2.8479 | 157.4698 |
| LG5 | LG5_2052@O7 | **TRP_480@H** | TRP_480@N | 3097 | 0.6194 | 2.8605 | 154.7983 |
| LG5 | LG5_2052@O | **ASN_764@HD21** | ASN_764@ND2 | 2633 | 0.5266 | 2.8573 | 154.6228 |
| LG6 | LG6_2052@O6 | TYR_411@HH | TYR_411@OH | 2571 | 0.5142 | 2.7355 | 161.1770 |
| LG4 | THR_701@OG1 | LG4_2052@H12 | LG4_2052@O11 | 2551 | 0.5102 | 2.7759 | 160.2375 |
| LG5 | LG5_2052@O3 | TYR_1876@HH | TYR_1876@OH | 2520 | 0.5040 | 2.7033 | 164.0507 |
| LG6 | **GLN_1349@O** | LG6_2052@H8 | LG6_2052@N8 | 2315 | 0.4630 | 2.8249 | 157.6969 |
| LG5 | LG5_2052@O6 | **ASP_476@H** | ASP_476@N | 2099 | 0.4198 | 2.8996 | 162.2650 |
| LG1 | TYR_405@OH | LG1_2052@H9 | LG1_2052@N8 | 2092 | 0.4184 | 2.8839 | 153.6789 |
| LG2 | LG2_2052@O9 | **TRP_765@HE1** | TRP_765@NE1 | 1966 | 0.3932 | 2.8411 | 157.0864 |
| LG2 | LG2_2052@O2 | **ARG_786@HH11** | ARG_786@NH1 | 1844 | 0.3688 | 2.8374 | 159.5531 |
| LG5 | **SER_479@OG** | LG5_2052@H6 | LG5_2052@N7 | 1633 | 0.3266 | 2.9118 | 161.5781 |
| LG1 | LG1_2052@O10 | **ASN_774@HD22** | ASN_774@ND2 | 1547 | 0.3094 | 2.8279 | 155.7230 |
| LG5 | PRO_474@O | LG5_2052@H13 | LG5_2052@N10 | 1536 | 0.3072 | 2.8748 | 162.6973 |
| LG6 | LG6_2052@O7 | **ASN_1350@HD22** | ASN_1350@ND2 | 1446 | 0.2892 | 2.8369 | 157.7615 |
| LG2 | LG2_2052@O13 | **ASN_787@HD22** | ASN_787@ND2 | 1441 | 0.2882 | 2.8343 | 160.9108 |
| LG1 | **GLN_1056@O** | LG1_2052@H4 | LG1_2052@N5 | 1390 | 0.2780 | 2.8645 | 152.7584 |
| LG6 | **ASP_1064@O** | LG6_2052@H2 | LG6_2052@N3 | 1345 | 0.2690 | 2.8795 | 161.8260 |
| LG6 | HID_730@NE2 | LG6_2052@H6 | LG6_2052@N7 | 1224 | 0.2448 | 2.9303 | 161.9641 |
| LG1 | LG1_2052@O13 | TYR_405@HH | TYR_405@OH | 1208 | 0.2416 | 2.7002 | 163.0822 |
| LG2 | LG2_2052@O10 | **TRP_480@H** | TRP_480@N | 1180 | 0.2360 | 2.8720 | 160.2672 |
| LG5 | **GLN_763@OE1** | LG5_2052@H16 | LG5_2052@N11 | 1170 | 0.2340 | 2.8598 | 158.7023 |
| LG5 | LG5_2052@O | **TRP_765@HE1** | TRP_765@NE1 | 1155 | 0.2310 | 2.8681 | 154.3362 |
| LG1 | **ASP_769@OD1** | LG1_2052@H14 | LG1_2052@N10 | 1148 | 0.2296 | 2.8456 | 154.9871 |
| LG2 | **GLN_763@O** | LG2_2052@H5 | LG2_2052@N6 | 1139 | 0.2278 | 2.8629 | 152.9542 |
| LG3 | **ASN_1080@OD1** | LG3_2052@H2 | LG3_2052@N3 | 1089 | 0.2178 | 2.8649 | 159.0076 |
| LG6 | **TRP_1066@O** | LG6_2052@H2 | LG6_2052@N3 | 1035 | 0.2070 | 2.8077 | 149.4834 |
| LG1 | **TRP_773@O** | LG1_2052@H10 | LG1_2052@N9 | 1029 | 0.2058 | 2.8608 | 160.3863 |

*In bold is highlighted the presence of hydrogen bonds with important residues for hemolysin activity. In this study, we consider the whole protein (heptameric structure), consisting of seven monomers each containing 293 residues. Therefore, the number described here is calculated by the position of the hydrogen bond in the monomer added to the total number of residues of each monomer and its multiples. Example: GLN1056 is equivalent to GLN177 as 1056 = 177+293+293+293.
